# Beyond animal glue: Paleoproteomic analysis of paint binders and adhesives in ancient Egypt

**DOI:** 10.1126/sciadv.ady3618

**Published:** 2026-08-26

**Authors:** Clara Granzotto, Rebecca J. Stacey, Meaghan Mackie, Kate Fulcher, Fabiana Di Gianvincenzo, Neal Spencer, Cecilie Brøns, Luise Ørsted Brandt, Mia Broné, Carl Heron, Enrico Cappellini

**Affiliations:** ^1^ Globe Institute, Faculty of Health and Medical Sciences, University of Copenhagen, Øster Farimagsgade 5-7, 1353 Copenhagen, Denmark.; ^2^ Department of Scientific Research, The British Museum, Great Russell St, London WC1B 3DG, UK.; ^3^ Novo Nordisk Foundation Center for Protein Research, University of Copenhagen, Blegdamsvej 3b, 2200 Copenhagen, Denmark.; ^4^ Department of Ancient Egypt and Sudan, The British Museum, Great Russell St, London WC1B 3DG, UK.; ^5^ Ny Carlsberg Glyptotek, Dantes Plads 7, 1556 Copenhagen, Denmark.; ^6^ Statensmuseerförvärldskultur/National Museums of World Culture, Box 5306, 402 27 Göteborg, Sweden.

## Abstract

Despite the wealth of artworks from ancient Egypt in collections worldwide, the number of analytical studies on the organic media used in paints and adhesives is limited, especially because microsamples are often required. In this study, we used mass spectrometry–based proteomics to confidently identify the species and tissues of origin of proteinaceous materials in painted ancient Egyptian artifacts from several collections dating 1425 BCE to 400 CE. Proteomics confirmed the use of glue prepared from the collagen of multiple animals and revealed the presence of plant proteins, including sesame and moringa products, previously undocumented in ancient Egyptian artworks. This prompts additional discussions about the origins of these materials, their symbolic and religious meaning in connection with deities, and their value within the artistic, cultural, and economic contexts of ancient Egypt. Our study demonstrates that combining paleoproteomics results with other analytical methods, archaeological evidence, and textual sources sheds light on expected and more unconventional organic materials in painted artworks from ancient Egypt.

## INTRODUCTION

A close examination of artifacts and an investigation of historical sources indicate that paint binders and adhesives used in ancient Egypt are mainly water soluble, with the exception of the attested use of beeswax in several Romano-Egyptian mummy portraits (*1*–*4*). The most common sources of these binders and adhesives are proteins (animal glue and egg) and polysaccharide-based (plant gum) materials (*5*, *6*). Often based on visual observations, animal glue was believed to be the most common proteinaceous artistic material used from a very early period in ancient Egypt (*7*). This material is made by boiling animal bones and connective tissues to retrieve collagen and other proteins. The likely animal sources for these applications can be indirectly identified from zooarchaeological studies, iconography, and texts (*8*–*10*), which provide information about animal introductions, domestication, and exploitation in ancient Egypt. However, the analytical characterization of the biological origin and chemical degradation of organic materials used to produce cultural heritage objects is important because it can provide direct empirical evidence about ancient artists’ techniques, material availability, and local exploitation of natural resources. While this approach is helpful in the development of conservation treatments, recent studies also proved that it can help correlate unconventional artistic materials with local culture and practices (*11*).

The presence of protein-based materials in ancient Egyptian painted objects has previously been established by solubility tests or Fourier transform infrared (FTIR) microspectroscopy (*12*). FTIR is a valuable screening tool to determine the presence of a proteinaceous material, as the sample can be collected and reused for other more specific techniques. The detection of proteinaceous material is based on the observation of characteristic bands such as amide I (1650 cm^−1^), amide II (1540 cm^−1^), amide II overtone (3070 cm^−1^), and N–H stretching bands (3300 cm^−1^) (*13*). However, this approach cannot provide the confident identification of what groups or classes of proteins are present in an artistic material, as they all show similar FTIR spectra that can also be affected by degradation and interference from other organic and inorganic substances, such as pigments. Contrary to FTIR, other analytical techniques, such as gas chromatography coupled to mass spectrometry (GC-MS) (*14*–*18*) and high-performance liquid chromatography (*5*, *19*), allow the three main proteinaceous source materials used in cultural heritage production (animal glue, egg, and casein) to be distinguished among and have been broadly applied to the investigation of microsamples from ancient Egypt. These approaches use total acid hydrolysis to reconstruct the most abundant proteins in the sample based on relative amino acid content percentages and on the presence/absence of specific markers, such as hydroxyproline for animal glue (*20*). This approach, however, is less suited to identify protein mixtures or proteins from other non-predefined source materials. Pyrolysis-GC-MS (Py-GC-MS) is another valuable technique to screen for the presence of proteinaceous materials based on the detection of nitrogen-containing cyclic compounds (diketopiperazines) (*21*). This technique requires minimal sample pretreatment, but results in complex compound modifications that make data interpretation challenging. In contrast, paleoproteomics, i.e., the application of untargeted mass spectrometry–based proteomic methods to ancient samples (interchangeably used with the word proteomics throughout the text), provides a more confident identification of non-predefined proteins in cultural heritage objects, as well as accurate information about their taxonomic origin (*22*–*26*), molecular alterations, and damage (*27*).

The application of proteomics to cultural heritage objects is growing, and guidelines have been published to assist heritage scientists with the interpretation of its results (*28*–*32*). So far, however, only a few artworks from ancient Egypt, namely, Romano-Egyptian panel paintings and gilded mummy masks (*33*–*37*), have been studied with this approach. Here, we use it to characterize a broad set of artworks from ancient Egypt to gain a better insight into the use of protein-based materials as both paint binders and adhesives. We have purposely selected and investigated artworks from different museums’ collections and time periods, and with different kinds of support and material use, for example, paint versus adhesive, with the aim of getting a broader understanding of the artists’ materials selection and application. In addition, objects that have been previously investigated with other analytical techniques were deliberately selected with the aim of comparing and complementing results obtained with different approaches.

**Fig. 1.**
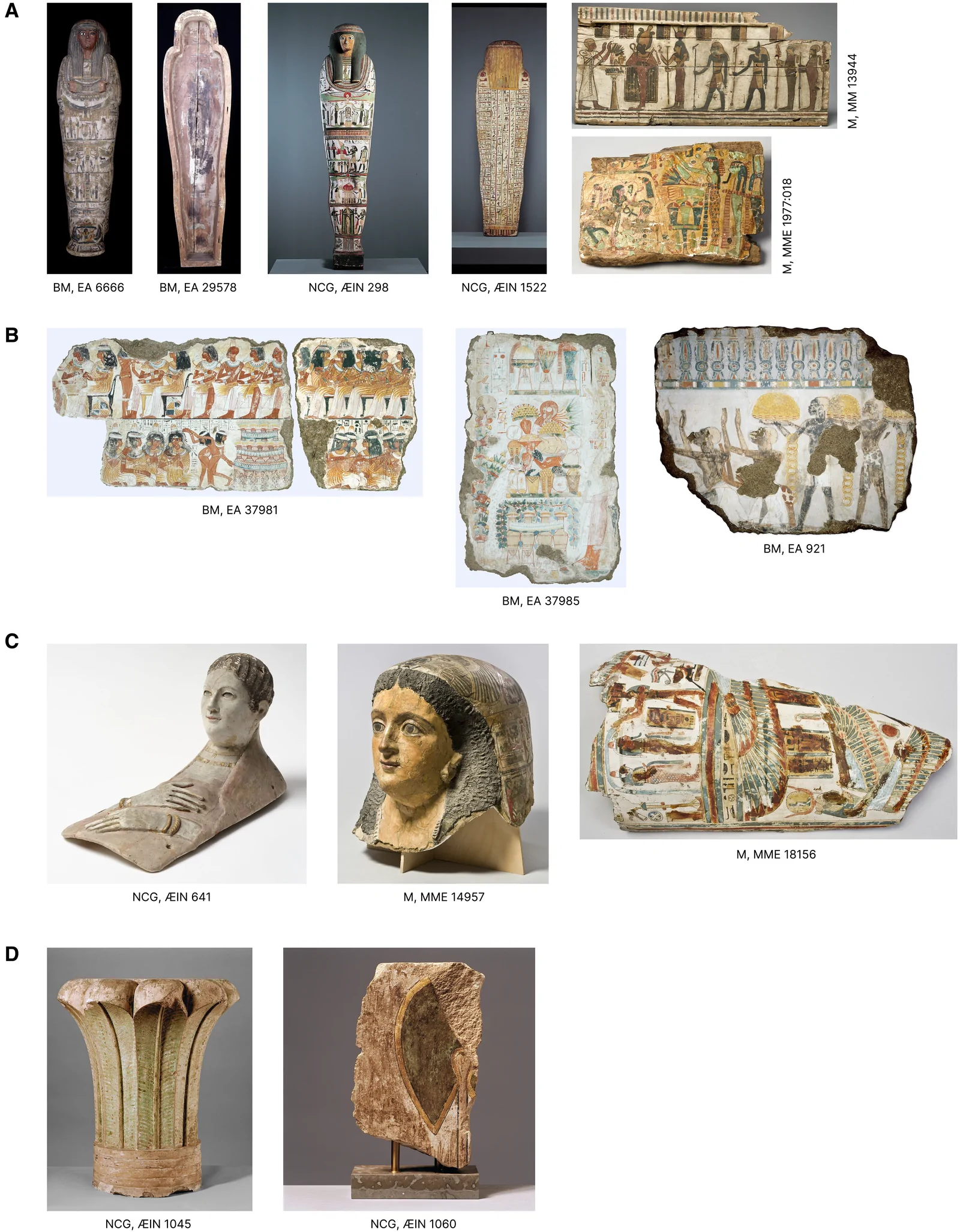
Artworks analyzed. Artworks analyzed in this study, with corresponding museum of origin (M: Medelhavsmuseet; BM: British Museum; NCG: Ny Carlsberg Glyptotek) and accession number, categorized by substrate typology: (**A**) wooden coffins (lid and case) and coffin fragments, (**B**) fragments of wall paintings (mud and plaster support), (**C**) mummy bust and cartonnage (painted plaster), and (**D**) column capital and fragment of a relief (painted limestone). More details of each artwork, together with the sampling spots, are provided in figs. S1 to S14. Photo credit: Medelhavsmuseet, © The Trustees of The British Museum, and Ny Carlsberg Glyptotek.

In total, 28 samples from 14 different painted artworks, dating 1425 BCE to 400 CE, from the Egyptian collections of the Medelhavsmuseet in Stockholm (Sweden), the British Museum in London (UK), and the Ny Carlsberg Glyptotek in Copenhagen (Denmark), were analyzed by shotgun proteomics using nano-flow liquid chromatography tandem mass spectrometry (nanoLC-MS/MS), following a first screening by FTIR analysis (see the Supplementary Materials). The set of selected artworks includes supports of various nature: wooden coffins (painted and with residues of the sealing adhesive), wall painting fragments from the tomb of Nebamun and Sobekhotep, painted plaster as applied in cartonnage construction as well as non-cartonnage objects, and painted limestone architectural elements (Fig. 1).

The primary goal of our research is to provide solid analytical evidence supporting the identification of the biological species and tissues of origin of the protein-based materials tested. The chemical damage of the proteins, represented by spontaneous and irreversible post-translational modifications (PTMs), was also examined to assess whether the adopted approach can distinguish original ancient materials from those deriving from later restoration or contamination, and possibly explain their origin. The overall results are critical in providing foundational knowledge of individual and regional painting techniques and material preferences. Together with complimentary historical and archaeological documentation, as well as future studies of a larger number of objects, these insights could contribute to enriching our knowledge of resource availability and their use through time.

## RESULTS

### Animal proteins

Proteomic analysis revealed the presence of collagen proteins from the subfamily Bovinae [collagen alpha-1(I)/COL1α1, collagen alpha-2(I)/COL1α2, collagen alpha-2(II)/COL2α2, and collagen alpha-1(III)/COL3α1] in the majority of the samples analyzed (Table 1). These proteins are indicative of the use of animal glue as an adhesive and/or a binding medium in both preparatory and paint layers. As reported in table S2, several diagnostic peptides allowed identification of the genus *Bos* as the source of the glue in most samples, and sometimes specifically of the species *Bos taurus* (cattle). Among the samples analyzed from paint, one from a wooden coffin green paint layer (MM 13944, sample S7) revealed a complex composition characterized by the presence of collagen proteins from *B. taurus*, Caprinae subfamily, although without achieving discrimination between *Ovis aries* (sheep) and *Capra hircus* (goat), and *Equus* species, with species-specific peptides from both *Equus asinus* (donkey) and *Equus caballus* (horse) (Table 1). Multianimal glues composed of Caprinae/Cephalophinae or Alcelaphinae (antelopes) collagens, mixed with collagen from *Bos* and *Equus*, were likewise found in the ground layer of a painted wooden coffin (ÆIN 1522), in a gilding sample from a plaster mummy bust (ÆIN 641) and in an adhesive applied most likely to seal the lid and the case of a wooden coffin (EA 6666, sample S2). Tryptic peptides of COL3α1 were identified in both adhesive samples analyzed in this study (samples S2 from EA 29578 and EA 6666), as well as in other paint and ground layer samples. COL3α1 is found in connective tissues such as the skin, intestine, and lungs (*38*), and its identification indicates that a specific type of glue, named hide glue, prepared at least in part using some or all of these tissues, was used in these samples (*39*, *40*).

**Table 1.** Proteomics results for materials of animal origin. Summary of the samples containing proteins of animal origin: museum’s collection, accession number (no.), period; sample number and description; animal of origin. “✓✓” and “✓” indicate species and genus identifications, respectively. *Bos* species include *Bos taurus* (cow), *Bos indicus* (zebu), and *Bos mutus* (yak). *Bos mutus* was excluded, even for matching sequences, because it is not plausible according to the geographical origin of the objects. Caprinae includes *Ovis aries* (sheep) and *Capra hircus* (goat). Alcelaphinae includes *Connochaetes taurinus* (blue wildebeest) and *Damaliscus lunatus* (sassaby), and Cephalophinae includes *Philantomba maxwellii* (Maxwell’s duiker). *Equus* genus includes *Equus caballus* (domesticated horse), *Equus asinus* (donkey), and *Equus przewalskii* (Mongolian wild horse), the last of which is excluded for geographical reasons. “?”: undetermined animal origin. !: possible contaminant. For more details on the taxonomic attribution, see table S2.

Museum, accession no., period	Sample description	Animal origin						
		*Bos taurus*	*Equus caballus*	*Equus asinus*	Caprinae	Alcelaphinae/Cephalophinae	*Oryx*	*Gallus gallus*
M, MM 13944, Greco-Roman*	S1—Ground layer	✓						
	S3—Brown paint	✓						
	S4—Red paint	?						✓✓
	S6—Red paint	✓						?!
	S7—Green paint	✓✓	✓✓	✓✓	✓			
M, MM 14957, Greco-Roman*	S1—Ground layer	✓						
	S3—Green paint	?						?!
M, MM 18156	S1—Ground layer	✓✓						
	S2—Blue paint	✓						
	S2—Ground layer	✓✓						
M, MME 1977:018, 21st†–22nd dynasty	S1—Ground layer	✓						
BM, EA 6666, 22nd dynasty‡	S2—Adhesive	✓✓	✓✓	✓✓	✓			
	S6—Blue paint	✓						
	S7—Ground layer	✓						
	S7—Green paint	✓						
BM, EA 37985, 1350 BCE	S1—Green paint							✓✓
BM, EA 29578, 22nd dynasty‡	S2—Adhesive	✓✓						
NCG, ÆIN 1522, 25th§–26th¶ dynasty	S2—Ground layer	✓✓	✓	✓	✓		✓	
NCG, ÆIN 298, 900–700 BCE	S2—Ground layer	✓						
NCG, ÆIN 641, 200–235 CE	S1—Gilding	✓			✓			
	S3—White paint	✓						✓✓
NCG, ÆIN 1045, 26th dynasty¶	Green inner paint	✓						
NCG, ÆIN 1060, 26th dynasty¶	White surface layer	✓✓						
	Yellow paint	✓						

* 332 BCE to 395 CE.

† 1085 to 950 BCE.

‡ 950 to 730 BCE.

§ 751 to 656 BCE.

¶ 664 to 525 BCE.

For all the historical materials, the level of deamidation of asparagine (Asn) and glutamine (Gln), often used to attest ancient protein authenticity (*41*, *42*), was considerably higher than the one measured in modern animal glue (fig. S15 and table S3). This is reassuring as animal glue is a common adhesive used for consolidation during conservation procedures; therefore, caution must be exercised when establishing a glue as original. Further discussion on the topic is reported in the Supplementary Materials.

Besides collagens, egg proteins were also identified in some of the analyzed objects (Table 1). Proteins specific to both egg white (ovalbumin and lysozyme) and egg yolk (vitellogenin-I and -II) were confidently identified in a green paint layer sample from the 18th Dynasty (approximately 1350 BCE) Tomb of Nebamun wall painting (EA 37985, sample S1) (fig. S7), suggesting the use of whole egg as the paint binder. Specifically, several peptides for each protein could be assigned to *Gallus gallus* (chicken) (see table S2 for the species-specific peptides). While we cannot exclude that egg proteins could come from contamination or previous conservation applications and more research on the same object is needed, the identification of an egg-based paint in our study is plausible and consistent with the materials available at that time (see the Supplementary Materials). Individual egg white proteins, specifically ovalbumin, ovotransferrin, and lysozyme, sometimes ascribable to the species *G. gallus* (Table 1 and table S2), were also identified in samples from other objects (table S1). The detection of these egg white proteins alone tentatively indicates the presence of only egg white. In contrast, the absence in samples MM 13944_S6 and MM 14957_S3 of ovalbumin, the most abundant protein in egg white, leads us to conservatively consider these egg traces as due to possible contamination.

### Plant proteins

**Fig. 2.**
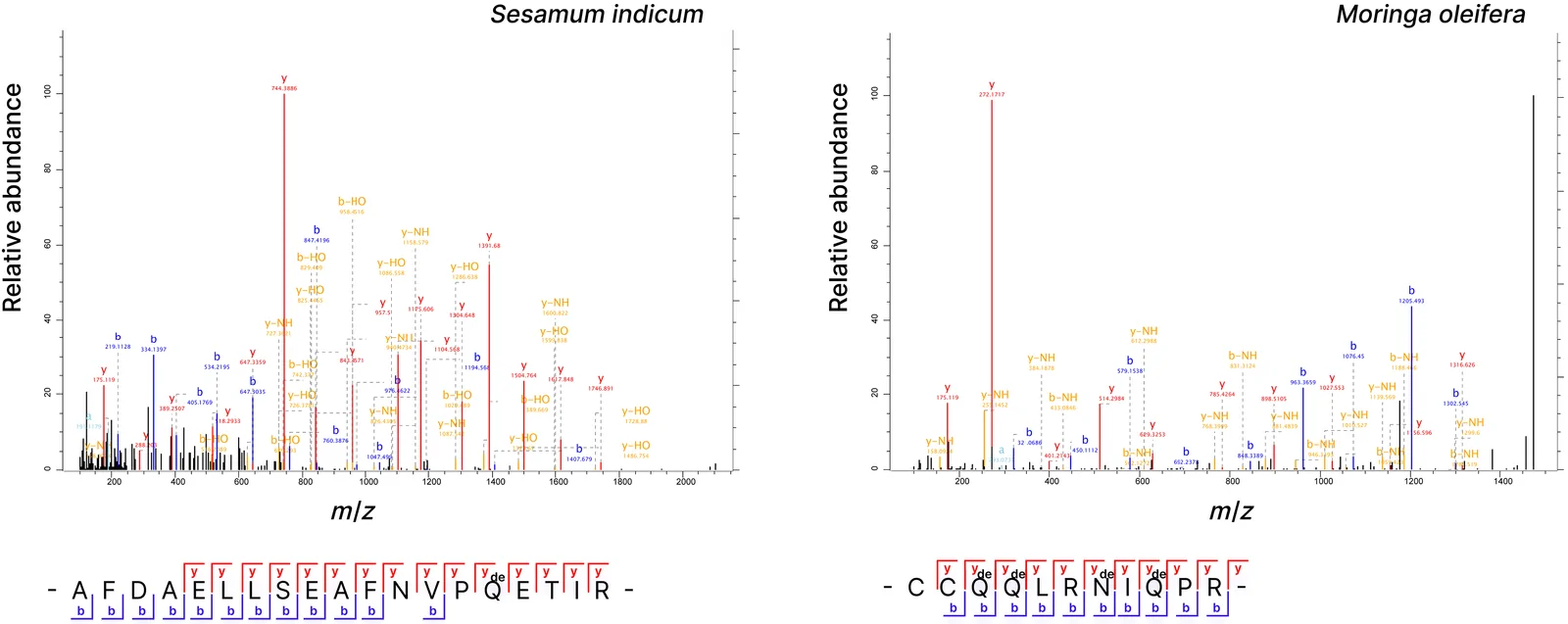
MS/MS spectra from seed-storage proteins. MS/MS spectra of peptides from 11S globulin seed-storage protein 2 from *Sesamum indicum* (left) and of 2S albumin from *Moringa oleifera* (right), both identified in the red paint layer sample S2 from MM 13944.

Unexpectedly, an initial search against the entire SwissProt database (*43*), followed by a second one using a database restricted to sequences exclusively from land plants (Embryophyta), supported the confident identification of plant proteins in four different artworks (Table 2). Seed-storage proteins specific to sesame (*Sesamum indicum*) and moringa, or drumstick tree (*Moringa oleifera*) were identified in two different artworks: (i) in the green paint layer from an earlier wall painting fragment from the tomb of Nebamun (1350 BCE, British Museum, EA 37981) and (ii) in the red paint layer from a Greco-Roman wooden coffin (332 BCE to 395 CE, Medelhavsmuseet, MM 13944). Specifically, in both samples, we observed four isoforms of 11S globulins from *S. indicum*, supported by a total of 35 peptides and 108 MS/MS spectral matches, and 37 peptides and 44 MS/MS spectra matches, respectively; while in the wooden coffin red paint sample, we additionally identified 2S albumin peptides uniquely attributed to *M. oleifera*, supported by 21 matching peptides and 220 MS/MS spectral matches (Fig. 2). The identified proteins and taxonomic specific peptides are reported in tables S1 and S2, respectively.

**Table 2.** Proteomics results for materials of plant origin. Summary of samples containing proteins of plant origin: museum’s collection, accession number (no.), period; sample number and description; plant of origin SI = *Sesamum indicum*; MO = *Moringa oleifera*; Tr = *Triticum aestivum*, *Triticum urartu*, *Triticum turgidum* subsp. *durum* and *Triticum dicoccoides*; Ho = *Hordeum vulgare* and *Hordeum vulgare* subsp. *vulgare*; SC = *Secale cereale*; SC (?) = possible *S. cereale*; taxonomic specific proteins with UniProt identifier, protein’s function. For more details on the taxonomic attribution, see table S2.

Museum, accession no., period	Sample description	Species/Genus	Taxonomic specific proteins (UniProt identifier)	Function
M, MM 13944, Greco-Roman	S2—Red paint	SI	11S globulin seed-storage protein 2 (Q9XHP0)	Seed-storage protein
		SI	Legumin B-like (A0A8M8USH5)	Seed-storage protein
		SI	11S globulin isoform 3 (Q2XSW7)	Seed-storage protein
		SI	11S globulin (Q9AUD2)	Seed-storage protein
		MO	2S albumin (W5S2K0)	Seed-storage protein
		MO	2S albumin (W5S2D2)	Seed-storage protein
		MO	2S albumin (W5S743)	Seed-storage protein
BM, EA 37985, 1350 BCE	S1—Green paint	Tr	Protein synthesis inhibitor II–like	Plant defense
		Tr	Low–molecular weight glutenin subunit	Seed-storage protein
	S2—Blue paint	Tr	Alpha amylase/trypsin inhibitor (fragment)	Plant defense
BM, EA 37981, 1350 BCE	S3—Green paint	SI	11S globulin seed storage protein 2 (Q9XHP0)	Seed-storage protein
		SI	Legumin B–like (A0A8M8USH5)	Seed-storage protein
		SI	11S globulin isoform 3 (Q2XSW7)	Seed-storage protein
		SI	11S globulin (Q9AUD2)	Seed-storage protein
BM, EA 921, 1425–1400 BCE	S5—Blue paint	Tr	Alpha-1,4 glucan phosphorylase	Sugar metabolism
		Tr	Putative major allergen PhI p 5	Plant defense
		Tr	Pollen allergen Dac g 3–like	Plant growth
		Tr	Sucrose-phosphate synthase (fragment)	Sugar metabolism
		Tr	l-Ascorbate peroxidase	Plant defense
		Ho	Pollen allergen KBG 41	Plant growth
		Ho	UTP-glucose-1-phosphate uridylyltransferase	Sugar metabolism
		SC	Pollen allergen Sec c 5 (F4MJM3)	Plant growth
		SC (?)	Exopolygalacturonase (G9I6F6)	Sugar metabolism
		SC (?)	Group 11 grass pollen allergen F7–1 (G9I6E7)	Plant growth
		SC (?)	Profilin (G9I6G4)	Plant growth

Additional catalytic proteins originating from cereals were identified in paint samples from the two wall painting fragments EA 37985 and EA 921 (Table 2). The majority of these proteins derived from the wheat genus (*Triticum*), with proteins specific to species such as bread wheat (*Triticum aestivum*), red wild einkorn wheat (*Triticum urartu*), emmer wheat (*Triticum dicoccoides*), and subspecies like durum wheat (*Triticum turgidum* subsp. *durum*), together with two proteins from barley (*Hordeum vulgare* and *H. vulgare* subsp. *vulgare*) and one protein from rye (*Secale cereale*) (tables S1 and S2).

The absence of sesame, moringa, and cereal protein traces in the blank negative controls prepared and analyzed alongside the ancient paint samples excludes the idea that these peptides derive from laboratory contamination. The detection of all the endogenous plant material is supported by the reliable identification of proteins from each cereal species and of multiple seed-storage proteins from sesame and moringa, all confidently recognized by a large number of taxonomic-specific peptide sequences matching multiple high-quality MS/MS spectra. With regard to sesame and moringa proteins, exogenous origin due to conservation treatment is unlikely due to the unusual source of the material identified. In addition, the Asn and Gln deamidation rates, measured for the seed-storage proteins in the red sample from the wooden coffin fragment MM 13944, were compatible with high degradation, therefore consistent with an ancient material (fig. S16). Deamidation rates for the plants’ origin proteins are discussed in the Supplementary Materials. The nature of these diverse plant proteins and the reason behind their presence in a variety of objects from ancient Egypt is further developed in the Discussion.

## DISCUSSION

This study presents the first extensive paleoproteomics-based investigation of the biological origin of proteinaceous materials used as binders and adhesives in ancient Egypt. Until now, paleoproteomics has established cow (*B. taurus*) as the primary resource for glue preparation in paint samples from ancient Egyptian artifacts (*33*, *35*), and only recently, studies of Romano-Egyptian mummy masks have identified animal glue prepared from *E. asinus* and a mix of animals (cattle, hippo, warthog, and wild boar) as binder and adhesive for gilding, respectively (*36*, *37*).

The analysis of several microsamples in our study has confirmed the extensive use of cow glue, and has in addition proven the use of animal glue from multiple mammalian species as the binding medium in a variety of objects (Table 1). COL1α1 peptides specific to the species *B. taurus* were identified, while in other instances, peptides were inferred to *B. taurus* as the most likely taxonomical origin among other *Bos* species identified—*Bos mutus* (yak) is not a plausible species for the geographical area, and there are still debates on the presence of *Bos indicus* (zebu) in ancient Egypt (*44*) (table S2). Sheep (*O. aries*) and goat (*C. hircus*) could not be distinguished in our results and were assigned to the subfamily Caprinae. Both species are plausible as they have been domesticated in Egypt since the Neolithic period (ended approximately 5000 BCE) (*45*). Besides some COL1α1 peptides uniquely assigned to the subfamily Caprinae, other COL1α2 and COL3α1 peptides are also shared with some antelopes from the subfamilies Cephalophinae and Alcelaphinae, which include the species *P. maxwellii* (Maxwell’s duiker), and *C. taurinus* (blue wildebeest) together with *D. lunatus* (sassaby) as the only plausible species, respectively; and the genus *Oryx* (*46*) (Supplementary Materials and table S2). Wild animals were likely hunted to provide meat and other animal products (*47*, *48*), and the unused parts could have been recycled in the glue production. Collagens from *E. caballus* (domesticated horse) and *E. asinus* (donkey) were also identified. When the two species could not be distinguished, the taxonomical origin is described as *Equus*. The use of another equine ancestor not currently sequenced cannot be excluded. Domesticated horses were introduced in Egypt in the Second Intermediate period (1650 to 1550 BCE) (*49*), and evidence of the domestic donkey in Egypt goes back to 3045 BCE (*50*), which is consistent with the dates of the artifacts investigated. While donkeys were used for agricultural work and their meat consumption can be assumed, horses were mainly a status symbol and the authors are not aware of zooarchaeological examples displaying specific butchery marks that might indicate horse meat consumption. Parts of, possibly, naturally dead horses could have been used to prepare glues, but more data are necessary to support this theory.

In addition to the animal sources, paleoproteomics provided empirical evidence on the tissue specificity of the proteins recovered. The detection of COL3α1 proteins indicated that glues were prepared by boiling animal skin, possibly also with connective tissues and cleaned bones. Compared to the glue obtained from just bones (bone glue), hide glue is described to be stronger (*51*) and was therefore expected to be found primarily in the adhesive samples. However, COL3α1 was also identified in paint and ground layers’ samples. This supports the hypothesis that raw materials were most likely selected based on availability rather than artistic application. In this regard, in our sample set, no notable correlation was observed between the choice of the animal species/part and the object type, the nature of the application, the color, the chronology, the geographical origin, or the use context (funerary or palatial). Paleoproteomics applied to a variety of artworks was therefore instrumental to get a better understanding of the animal species availability and to confirm glue production as a valued by-product of the butchery of animals for meat consumption or other animal-related processes.

Besides the detection of collagen proteins, paleoproteomic analysis provided the unexpected but confident identification of distinctive seed-storage proteins from sesame (*S. indicum*) and moringa (*M. oleifera*) (Table 2). Both plant genera were available in ancient Egypt at the time of the objects investigated in our study [(*52*, *53*) and the Supplementary Materials]. Understanding the preparation, use, and meaning of this plant material, which, to the authors’ knowledge, has never been identified before in painted artworks, is intriguing.

**Fig. 3.**
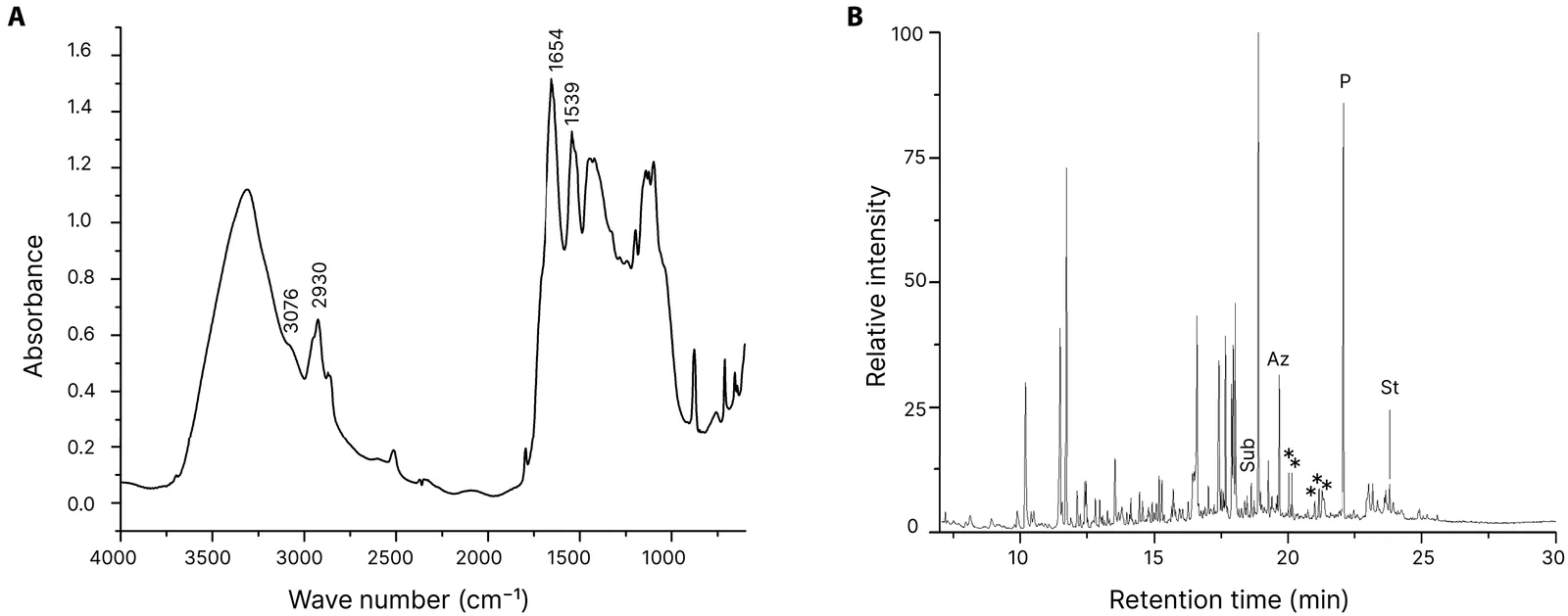
FTIR and Py-GC-MS data. (**A**) FTIR spectrum and (**B**) Py-GC-MS total ion chromatogram of the red sample S2 from the wooden coffin fragment MM 13944. Sub, suberic acid; Az, azelaic acid; P, palmitic acid; St, stearic acid; asterisks, diketopiperazines.

Seed-storage proteins from *S. indicum* like the ones identified in our study (table S1), make up to 60 to 70% of the proteins in sesame seeds (*54*) and have been previously associated with the production of oils for illumination in China (*55*). In ancient Egypt, different plants such as castor, almond, linseed, olive, radish, safflower, and many others were possible sources of vegetable oils, used for a variety of purposes: cooking, illumination, personal toilette, medicine preparation, mummification, and religious ceremonies (*47*, *56*–*59*). Oils extracted especially from sesame and moringa seeds were of particular high quality, and used for cooking, medicine, mummification (*7*, *60*), insect repellent (*61*), and cosmetics (*62*, *63*). While GC-MS and Py-GC-MS analysis identified oils from undetermined plant species as the binding medium in paint samples from ancient Egypt in other studies (*4*, *64*), the use of sesame and moringa oil as paint binder is unlikely as these plants’ seed would produce a semidrying and non-drying oil, respectively (*47*), which would not offer good paint binding properties. In addition, FTIR results of the red paint from the wooden coffin MM 13944 (sample S2) do not support the use of sesame/moringa oil, as the characteristic bands identifying the lipidic compound of a plant oil (prominent C═O stretching band around 1730 cm^−1^, and bands at 2920 and 2850 cm^−1^ from the asymmetric and symmetric CH stretching, respectively) (*65*) were not clearly observed in the FTIR spectrum. A possible shoulder in correspondence of the amide I band around 1720 cm^−1^, and a slightly more pronounced band at 2930 cm^−1^ can, at best, be indicative of the possible presence of lipid traces (Fig. 3A). Instead, FTIR analysis clearly indicates that proteins represent the major organic component (bands at 1654 cm^−1^ from amide I, 1539 cm^−1^ from amide II, and 3076 cm^−1^ from the amide II overtone). Similarly, Py-GC-MS analysis, a technique that is more sensitive to oils, waxes, and natural resins, clearly shows the presence of diketopiperazines, a nitrogen-containing cyclic compound associated with the presence of a protein-based material (*21*), along with fatty acids (palmitic, stearic, azelaic, and suberic) in a proportion suggesting the presence of a semi-/nondrying oil (Fig. 3B) (*66*).

The integration of all the evidence provided by proteomics, FTIR, and Py-GC-MS indicates that the organic component consists of a dominant protein-rich fraction associated with residual traces of semi-/nondrying oil. This material might represent a by-product of sesame and moringa seed processing. With regard to sesame, papyri mention the use of its seed as food, besides for making oil (*67*). We exclude the material to be a by-product of the direct processing of the whole seeds because neither polysaccharidic compounds nor lignins, such as sesamin and sesamol, were detected in the pyrogram (*68*). Another hypothesis is the use of a possible residue left after oil production. Little is known about oil manufacture in ancient Egypt. Cleaning, dehulling, and grinding of the seeds are assumed to be necessary steps, but it is still unclear whether the actual extraction happened via cooking in water or cold pressing, as papyrological evidence of “oil boiler” and “bag press” might refer to scented oils and not oils per se (*47*, *67*). Nevertheless, both extraction processes would have left a residue, or “seed cake” (*67*), that in the case of the cold pressing protocol would likely be rich in seeds’ proteins.

The sesame proteins detected in red sample S2 from the coffin fragment MM 13944 are the same ones identified in the Nebamun wall painting fragment EA 37981, suggesting the use of a similar plant material. In support of the hypothesis of the use of a protein-rich product of sesame seed also for this artwork, GC-MS analysis from a former study of a green sample, obtained in proximity of the sample taken from the wall painting, did not show fatty acids that would be indicative of an oil (*17*).

**Fig. 4.**
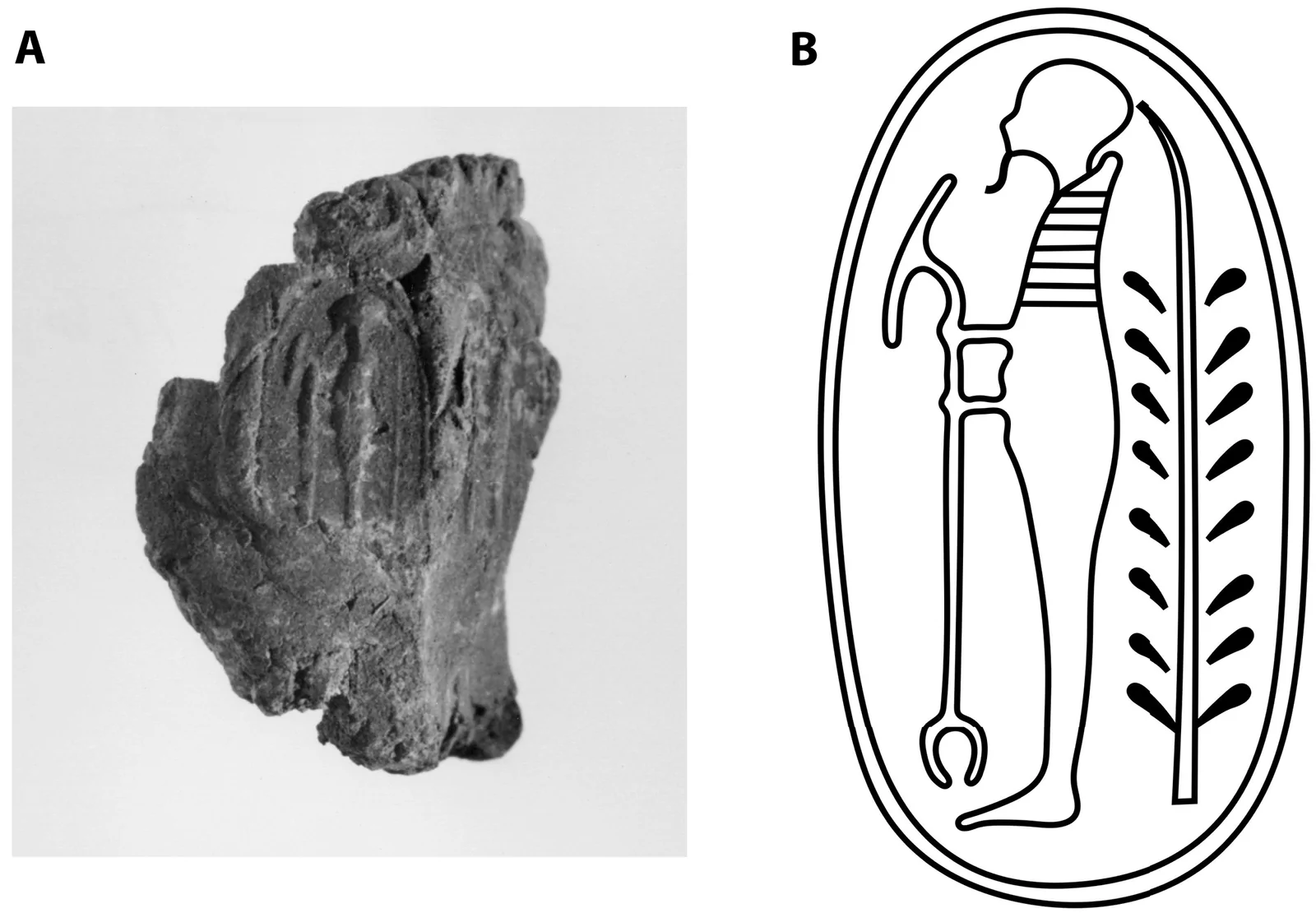
Document seal devoted to Ptah and its reproduction. (**A**) Document sealing, approximately 1390 to 1352 BCE, Upper Egypt (Thebes), mud, H. 2.7 cm (1 ^1^/_16_ in.); w. 2.2 cm (^7^/_8_ in.), The Met 12.180.654, Rogers Fund, 1911, 1912; (**B**) drawing of the seal after Hayes 1951 (*70*).

Intriguingly, the significance of the finding extends beyond the practical importance of the plant-based material availability and use. The moringa tree had a strong symbolism in ancient Egypt, as it was one of the manifestations of the god Ptah, one of whose epithets being “Ptah khery-bak-ef,” i.e., “Ptah Who is Under His Moringa Tree” (*69*), as shown in the only known representation of the moringa tree on a seal, dating 1390 to 1352 BCE (Fig. 4) (*70*). Ptah was believed to be the patron of craftsmen, representing the bridge between the first creative idea in the artist’s mind and the final product (*60*, *71*). The detection, on the coffin fragment, of a material produced from moringa might actually give a practical explanation to the symbolic association of the god Ptah with this plant, further corroborating the connection between Ptah and artisans. Using paleoproteomics, we clearly show that moringa is the precious source of the material the artisan needs to accomplish their creation. Alternatively, the use of moringa could represent a possible action taken by the artist to devote or safeguard the final product of their craftsmanship and creativity. To better support this suggestion, and to understand whether these sesame and moringa seeds’ protein-rich products were used as paint binders or applied in localized areas for different purposes, including during religious ceremonies, further analyses should be performed to systematically test their occurrence in painted artifacts. Regardless, to the best of the authors’ knowledge, this study represents the first molecular-based confirmation of the presence and use of materials from sesame and moringa species in ancient Egypt. Until now, the use of moringa tree products in the Greco-Roman period was supported only by textual interpretation and limited archaeobotanical findings (Supplementary Materials). The identification of the same sesame proteins in paint samples from objects belonging to different museum collections and chronological periods and made from different materials supports the possibility that, in ancient Egypt, sesame was used more diversely than previously thought. However, to better define all the possible uses of this plant in this region, further research, systematically focusing on many other classes of objects, will be required. Our study confirms that untargeted proteomics is fundamental to reveal the composition and source of expected, as well as unexpected, proteinaceous materials. Only the integration of proteomics with more established techniques such as FTIR and Py-GC-MS, and the interpretation of analytical results with knowledge on artists’ materials and their manufacture, as well as archaeological evidence, allowed us to propose the use of by-products from seeds manufacturing and, therefore, to expand our knowledge on materials availability, preparation, and symbolism.

Other plant proteins from cereals, primarily wheat, with the addition of proteins from barley and rye, were identified by proteomics in the paint layers from two different wall paintings (Table 2). Archaeological and archaeobotanical findings indicate that agriculture in Egypt was established around the 6th millennium BCE with emmer wheat (*T. turgidum* subsp. *dicoccoides*, a wild type of the domesticated *T. turgidum* subsp. *durum*) and hulled barley (*H. vulgare*) being the primary ingredients of two of the most important foods in the ancient Egyptian diet: bread and beer (*72*). The identification of wheat and barley in our study is consistent with what was available in ancient Egypt, and a more critical discussion of the exact species identified is described in the Supplementary Materials. With regard to *S. cereale*, only one protein from pollen grains, not present in seeds, was identified as species specific to rye, while three other proteins were attributed to *S. cereale* × *T. turgidum* subsp. *durum*, a modern crop non-existent in ancient Egypt (table S2). These proteins have not been sequenced yet for *S. cereale*; therefore, we cannot exclude their specificity to this plant. The identification of rye in the Tomb of Sobekhotep paint sample seems consistent with the time period as rye pollen grains were detected in lake deposits in the Fayium region (Northern Egypt) dating around 5000 BCE (*73*).

The reason behind the presence of cereal proteins is not straightforward. These cereal proteins might originate from sources such as (i) original or later contamination, and (ii) cereal products. With regard to the first possibility, cereal debris could have reached the tomb during the production of the wall paintings, for example, carried as food in baskets, coming from workers’ clothes, or present in the grinder(s) used to process the painting material, if used for both food and paint processes; or they could have come from a later contamination during excavation or conservation campaigns where food could have been consumed. With regard to the wall painting fragment from the Tomb of Sobekhotep (EA 921), this hypothesis seems unlikely because, among the 31 proteins detected in the paint sample, 8 are genus or species specific to mainly wheat, followed by barley and rye (Table 2 and tables S1 and S2), which would indicate the improbable accidental incorporation of multiple kinds of cereal products in the paint layers. Similarly, the finding of a seed-storage protein in the green paint sample from the wall fragment EA 37985 (Table 2) suggests the use of wheat grains (fig. S7). Considering the second option of origin of these proteins, aqueous solutions from wheat, such as flour and starch pastes, are used as glues and adhesives in painting production, as well as during conservation treatments (*15*, *74*). The proteome of wheat starch and flour pastes, as well as raw products, has been recently published (*75*). Some of the wheat proteins we identified are consistent with the ones published in the literature for raw flour and the flour paste water-soluble fraction, such as low–molecular weight glutenin subunit and alpha amylase/trypsin inhibitor, found in EA 37985, and sucrose-phosphate synthase and alpha-1,4 glucan phosphorylase, found in EA 921. Proteomics results suggest the intentional use of a flour product in the Nebamun wall painting fragment (EA 37985) as proteins from only the *Triticum* genus were identified, alone or mixed with chicken whole egg as observed in the green paint area (Tables 1 and 2). It is unlikely that the flour product originates from a restoration treatment because, while the Nebamun painting underwent a number of conservation campaigns, a previous study of these conservation materials excluded the presence of any plant-based product (*76*).

With regard to the other cereal proteins identified in the wall painting from the Tomb of Sobekhotep (EA 921), proteins such as pollen allergens are found in pollen grains and not seeds (*77*), which might suggest the incorporation, deliberate or not, of different plant components. In addition, some of the taxonomic specific proteins identified are involved in the metabolism of sugars (Table 2), but the use of any fermentation product from wheat and barley was excluded as no yeast proteins, among the ones sequenced and available online, were identified in the sample (when MS/MS spectra were searched against a restricted Saccharomycetaceae database). Therefore, similarly to the moringa and sesame discovery, it is plausible that we are dealing with a water-soluble material that was possibly a by-product of these cereals’ preparation processes. Further analyses and future studies will help to discern patterns and to arrive at a clearer understanding of the use of cereals products in paint layers.

The untargeted proteomics approach additionally allowed us to corroborate and complement unclear results from artworks that were previously investigated with GC-MS and Py-GC-MS techniques. Notably, paleoproteomics unveiled the use of a by-product of sesame seed processing in the Nebamun wall painting fragment EA 379815, when former GC-MS analysis could only detect the presence of a proteinaceous material, and it similarly identified chicken (*G. gallus*) whole egg together with a wheat-based material (Tables 1 and 2) in the fragment EA 37985 from the same wall painting, which adds data to the identification of animal glue by former GC-MS analysis (*17*). In addition, paleoproteomics challenged the previous GC-MS findings from a relief fragment of the Palace of Apries (ÆIN 1060) (*18*) by suggesting the use of animal glue in the paint layer, and provided further information on the species of origin of the glue, which was previously unknown.

In conclusion, this research emphasizes how valuable proteomics is for the identification of unconventional materials, which might be misidentified or unidentified by other analytical strategies simply because of the limited number of proteinaceous reference materials included in their databases. It also aims at stressing how reliance on a single analytical technique can be misleading and highlights how multianalytical approaches, interpreted in the context of wider archaeological evidence, allow reconstructing complex scenarios, getting a more holistic view of the object, and opening different doors for interpretation of materials preparation and choices. The results of this study provide a starting platform for exploring patterns in terms of chronology, regionalism, different workshops, and preferences for specific materials based on pigments and substrates in Ancient Egypt. A larger analytical dataset, further combined with the archaeological and textual accounts, would possibly offer answers to some of these still pending questions.

## MATERIALS AND METHODS

### Sample information

The samples analyzed in this study have been obtained from the Egyptian collections of 3 museums: the Medelhavsmuseet—Museum of Mediterranean and Near Eastern Antiquities (Stockholm, Sweden), the British Museum (London, UK), and the Ny Carlsberg Glyptotek (Copenhagen, Denmark). Objects were selected following several criteria: different typology (wooden coffins, cartonnage, wall painting, painted plaster, and limestone), availability of date/geographical area information, previous scientific studies, curatorial interest, and knowledge of the object conservation history. Information on the sampling technique is provided in detail in the Supplementary Materials, together with the complete list of objects investigated and the exact sampling spots (figs. S1 to S14).

### FTIR

At the Centre for Art Technological Studies and Conservation—CATS (Copenhagen, Denmark), FTIR analysis was performed with a Bruker Hyperion 3000 FTIR spectrometer with a Hyperion microscope [15× objective, mercury cadmium telluride (MCT) detector], while at the British Museum, analyses were performed using a Nicolet 6700 spectrometer with a Continuum IR microscope (Cassegrain 15× objective, MCT/A detector). A microportion of each sample was crushed in a diamond cell and analyzed in transmission mode. Acquisitions were performed in the range of 4000 to 600 cm^−1^ at a resolution of 4 cm^−1^. Each spectrum is the sum of multiple scans (128 to 512) according to the response of the different samples. The spectra data were processed using OPUS 7 (CATS) and OMNIC 8.3 (British Museum) software. Library databases (IRUG Edition 2000 and 2007) were used for spectra interpretations.

### Py-GC-MS

A portion (tens of micrograms) of the red sample (S2) from the wooden coffin fragment MM 13944 was derivatized by the addition of 1,1,1,3,3,3-hexamethyldisilazane (chemical purity 99.9%, Sigma-Aldrich, USA) before insertion into a Frontier Labs Multi-Shot PY-3030D pyrolyzer, with the furnace at 600°C. The pyrolyzer is interfaced to the gas chromatograph (Agilent 6890) coupled with a single quadrupole mass spectrometer (Agilent 5973). The analysis was carried out in splitless mode using a Frontier Lab Ultra Alloy 5 column (30 m by 0.25 mm by 0.25 μm). The inlet and MS transfer line were held at 300°C. The carrier gas was helium, used at a constant flow of 1 ml/min. The GC oven temperature program was as follows: 50°C for 1 min, ramped to 320 at 10°C/min, followed by a 20-min isothermal period. Acquisition was performed in scan mode [mass/charge ratio (*m*/z) 29 to 550]. Data analysis was performed on an Agilent MassHunter Qualitative 10.0 using the NIST 2017 spectral libraries.

### Protein extraction

Each collected sample was placed in an Eppendorf Protein Lo-Bind tube. One hundred microliters of an aqueous buffer containing guanidinium chloride (GuHCl) 2 M, tris(2-carboxyethyl)phosphine) (TCEP) 10 mM, chloroacetamide (CAA) 20 mM, and tris 100 mM was added to each sample and incubated for 3 hours at 80°C. After digestion for 2 hours at 37°C with 0.2 μg of rLysC (Promega, Sweden), samples were diluted to a final concentration of 0.6 M GuHCl by adding a solution of 25 mM tris in 10% acetonitrile (ACN) and lastly digested overnight under agitation at 37°C with 0.8 μg of Trypsin (Promega, Sweden). Digestion was quenched by acidifying the solution to pH 2 using 10% trifluoroacetic acid (TFA) (*27*). After centrifugation, the supernatant was recovered and the peptides were collected on in-house made C18 extraction stage tips (*78*). Stage tips were stored at −18°C before MS/MS analysis. A blank control, containing only the reagents for proteomics sample preparation, was analyzed together with each batch of samples.

### nanoLC-MS/MS

Because of two sessions of analysis, samples from the painted limestone ÆIN 1060 and ÆIN 1045 have a slightly different method than the others due to mass spectrometer availability and current methods at the time. For these two samples, peptides were eluted from the stage tips using 20 μl of 40% ACN and 0.1% TFA in water followed by 10 μl of 60% ACN and 0.1% TFA in water. ACN was removed by vacuum centrifugation at 45°C until a volume of approximately 3 μl of solution was left and resuspended with 5 μl of 0.1% TFA and 5% ACN before injection. These samples then follow the LC-MS/MS “Copenhagen” method published for ancient samples as used by Demarchi *et al.* (*79*). Peptides were separated on a 165-min gradient on a 50-cm column (75 μm inner diameter) in-house laser pulled and packed with 1.9-μm C18 beads (Dr. Maisch, Germany) on an EASY-nLC 1200 (Proxeon, Denmark) connected to a Q-Exactive HF mass spectrometer (Thermo Fisher Scientific, Bremen, Germany). Buffer A was 0.1% formic acid in milliQ water; buffer B was 0.1% formic acid in 80% ACN. The peptides were separated with increasing buffer B (up to 60% at 140 min), with a flow rate of 200 nl/min. Column temperature was maintained at 40°C using an integrated column oven. The Q-Exactive HF was operated in data-dependent top 10 mode. In short, MS parameters are as follows: MS1—120k resolution at *m*/*z* 200 over the *m*/*z* range 300 to 1750, an automatic gain control (AGC) target of 3e6, and a maximum injection time (IT) of 20 ms; MS2—60k resolution over the range of 200 to 2000 *m*/*z*, HCD collision, an AGC target of 2e5, a max IT of 108 ms, a normalized collision energy (NCE) of 25, an isolation window of 1.3 *m*/*z*, and a dynamic exclusion of 30 s. Spray voltage was 2 kV, S-lens RF level was at 50, and the heated capillary was set at 275°C.

For the remaining majority of the samples, the LC-MS/MS protocol instead followed that described by Mackie *et al.* (*27*), and the differences are as follows. Stage tips were eluted only with 30 μl of 40% ACN and 0.1% TFA to better maintain the column and avoid higher weight contaminants. Peptides were separated with a 77-min gradient on a 15-cm column, also prepared in-house as above. LC buffers were the same, and peptides were separated with increasing buffer B (up to 80% at 62 min), with a flow rate of 250 nl/min. While the nLC system was the same, these samples were analyzed by a Q-Exactive HF-X (Thermo Fisher Scientific, Bremen, Germany). Differences to the HF method are summarized here: MS1—*m*/*z* range 350 to 1400, a max IT of 25 ms; MS2—an NCE of 28, an isolation window of 1.2 *m*/*z*, and a dynamic exclusion of 20 s. For both LC-MS/MS methods, a wash-blank method using 0.1% TFA and 5% ACN was run before and between injections to prevent carryover.

### MS/MS data analysis

Raw data were processed with the MaxQuant software (*80*) version 1.6.1.0. The MS/MS spectra were searched against an in-house database containing publicly available sequences of the most common proteins found in artistic materials (collagens, egg, and milk proteins). A follow-up search was performed using a database including COL1α1 and COL1α2 sequences for several African Bovidae species (*46*), for samples containing collagen proteins associated with Caprinae. All spectra were also searched against a wider database (SwissProt database from UniProt, January 2017 and July 2025) (*43*) to search for proteins originating from other sources. Samples showing the presence of plant origin proteins when using the SwissProt database underwent a third search against a more restricted database including all landgreen plants (Embryophyta). On the basis of the results from the SwissProt and Embriophyta databases, the samples of interest were searched against more restricted databases containing all proteins sequenced for: (i) *S. indicum*; (ii) *M. oleifera*; (iii) *S. cereale*, *Triticum*, and *Hordeum* genera; and (iv) Saccharomycetaceae family (February 2026). The following modifications were set for the database searching: carbamidomethylation (C) as fixed modification; methionine (M) oxidation, deamidation of glutamine (Q) and asparagine (N), conversion of N-terminal Q and glutamic acid (E) to pyroglutamic acid (pyro-E), and hydroxyproline (for the collagen containing samples) as variable modifications. Trypsin was set as the digestion enzyme. Peptides were searched with maximum two missed cleavages, and minimum peptide length was set at 7. The minimum Andromeda score for both modified and unmodified peptides was set to 40 and protein false discovery rate (FDR) was set at 0.01. Measurement error was set to 4.5 and 20 parts per million (ppm) for MS1 and MS2, respectively. Contaminant proteins were assessed using the contamination.fasta provided by MaxQuant, which includes common laboratory contaminants, and omitted from further analysis. Proteins were confidently identified when at least two non-overlapping razor + unique peptides were observed. Peptides were considered species diagnostic when, after searching against the entire NCBI protein database using the pBLAST alignment tool (*81*), and against the Viridiplantae (green plants) database using the Phytozome BLAST tool (*82*), they could be assigned to a single species or to a limited number of species among which only one could be considered plausible according to the nature of the material analyzed (its geographic origin and dating). These species are nonetheless reported in table S2. When present, these non-plausible species are reported in table S2 in parenthesis. When species-specific peptides were not identified for a protein, the protein was attributed to the lowest possible taxonomy rank (to genus, subfamily, family, etc.). Following the parsimony principle, when a protein could not be attributed to a specific species or a meaningful taxonomy rank, it was assigned to the same species of proteins from the same sample, if the unspecific peptides were compatible with that species. The degree of deamidation was evaluated using a publicly available tool (https://github.com/dblyon/deamidation) described in Mackie *et al.* (*27*). The Dependent Peptides algorithm of MaxQuant (*83*) (FDR 0.01) was used to investigate the presence of other PTMs, and an additional search was performed adding some known variable modifications associated to photo-oxidation (*27*).

## Supplementary Materials

### The PDF file includes:

Supplementary Text Figs. S1 to S17 Legends for tables S1 and S2 Table S3 References
