# Supplementary material for: Beyond animal glue: Paleoproteomic analysis of paint binders and adhesives in ancient Egypt
Source: Sci Adv. 2026 Aug 26;12(35):eady3618. doi: 10.1126/sciadv.ady3618 (PMC13510678; doi:10.1126/sciadv.ady3618)
Supplement: Supplementary file 1 [file sciadv.ady3618_sm.pdf]

Science Advances
AAAS

Supplementary Materials for

Beyond animal glue: Paleoproteomic analysis of paint binders and adhesives in ancient Egypt

Clara Granzotto *et al.*

Corresponding author: Clara Granzotto, clara.granzotto@gmail.com; Enrico Cappellini, ecappellini@sund.ku.dk

Sci. Adv. **12**, eady3618 (2026)
DOI: 10.1126/sciadv.ady3618

The PDF file includes:

Supplementary Text
Figs. S1 to S17
Legends for tables S1 and S2
Table S3
References

Other Supplementary Material for this manuscript includes the following:

Tables S1 and S2

Supplementary text

Sampling, preliminary and supporting analyses

Micro-samples - estimated to be around few tens to hundreds of micrograms - from the artworks were taken using a surgical blade and needle using magnifying glasses. Samples were examined visually under the microscope and, if present, each layer (e.g. ground and paint layers) was manually separated and analysed. While precautions were taken to guarantee each sample to come from a specific layer, we cannot exclude the possibility of particle contamination from other layers, as well as migration of the organic material throughout the polychromy during its original application.

Before proceeding with micro-destructive proteomics analysis, a fraction of the micro-samples was screened for the presence of proteinaceous traces by FTIR, as the material can most time be retrieved and used for further analyses. Samples from the Nebamun wall painting (EA 37985, EA 37981), the Tomb of Sobekhotep (EA 921) and the wooden coffin fragment (MM13944, samples S4, S6 and S7) were not analyzed by FTIR but instead used in their entirety for proteomic analysis due to the extremely limited amount of sample available. Samples from architectural elements ÆIN 1045 and ÆIN 1060 were used in a previous session of proteomics analysis and were not screened by FTIR. The distinctive infrared spectrum of a proteinaceous material is characterised by the presence of two strong bands around 1650 and 1550 cm^{-1}, designated amide I and amide II respectively, together with the amide II overtone at 3070 $cm^{-1,}$ and the N-H stretching band at 3350 cm^{-1} (65). As an example, the infrared spectrum of the adhesive sample S2 from the coffin EA 29578 is shown in Fig. S6B. FTIR analysis provided for some samples additional information on the nature of the inorganic component, mixed with the protein-based material: calcite ($CaCO_3$) was detected in the ground (Fig. S3 left), and Egyptian blue and huntite, a magnesium/calcium carbonate, were identified in the blue paint sample from the same cartonnage (Fig. S3 right).

Deamidation and post-translational modifications study

The percentage of samples containing animal glue within the group of artworks investigated in this study suggests that glue was a major protein-based material used as a binding medium and adhesive in ancient Egypt. However, animal glue is also a common adhesive used for consolidation during conservation procedures. Therefore caution must be exercised when establishing a glue as original, since it might derive from a more recent, and sometimes undocumented, conservation treatment. An approach to evaluate protein damage which, in the conservation science field is often associated with the protein age, consists of calculating the deamidation level of protein residues (41, 42). Asparagine (Asn) and glutamine (Gln) spontaneously deamidate over time and the extent of this chemical alteration is considered a major indicator of protein deterioration (85, 42). While it is known that the process of preparing animal glue, which involves boiling animal parts for a long time, is a source of protein deamidation (86), its calculation is still a valuable tool to evaluate protein damage, especially if compared to modern reference animal glues prepared following a similar traditional way.

In our study the percentage of deamidation for Asn and Gln was calculated for the animal glue-containing samples - each consisting of one single paint or ground layer - using the total number of collagen proteins with a determined taxonomic origin, regardless of the species. The results, together with data from a modern glue reference material (possible rabbit skin glue

prepared in 1978 following the traditional method) (74), are shown in Fig. S15, and values are reported in Table S3. The deamidation level of the samples from ancient Egypt was substantially variable, ranging 36.5±21.0 to 100% for Asn, and 6.0±4.0 to 69.3±4.2% for Gln. For all samples, deamidation of Asn was always higher, sometimes double, than that of Gln. This behaviour is expected since Asn is known to deamidate faster, and therefore, the Gln deamidation level is considered more informative over longer time scales (85). The percentage of deamidation of Gln was higher than that of the reference modern glue for all samples analysed, which suggests the proteinaceous material in the art sample to be more degraded. Deamidation levels cannot however provide any further information on the material age as they are affected by several factors, including but not limited to: temperature, pH, relative humidity, material preparation process, animal parts used and the animal of origin (42, 87, 39). In our study, no correlation was observed between the level of deamidation and the date of the object, and the Gln deamidation level was sometimes inconsistent among samples obtained from the same work of art. This can be partly explained by the variable number of peptides recovered and used for the calculation, which was at times below the significant threshold for this calculation (<20) (88). Another explanation can be found in the use, within the same object, of animal glues prepared from one or multiple animal species, which have been proven to show different levels of deamidation, with mixed-species glues experiencing less deamidation (39). Therefore, when analysing collagen-containing samples from works of art, qualitative analysis might provide more information about the authenticity of a material. The identification of glue prepared from multiple animal species, such as some samples in this study, suggests the likely original nature of the glue. Modern glues are instead usually prepared using either cow or rabbit tissues.

The same approach was used to evaluate the preservation state of the sesame and moringa seed storage proteins (Fig. S16). The red paint sample from the wooden coffin MM 13944 showed a high level of deamidation for both Asn (60±7%) and Gln (77±4%), indicating a degraded proteinaceous material. The green paint sample from the Nebamun wall painting EA 37981 did not show any degree of deamidation, possibly due to the fact that the number of peptides that could undertake deamidation was close to the threshold (27 for Asn and 30 for Gln). It’s important to note that sesame and moringa seeds could have been subjected to high temperatures if boiled or heated during oil extraction or other seeds treatments (67), which would likely affect deamidation levels. Therefore, similarly to collagen proteins, deamidation results need to be supported by discussion on the nature of the proteinaceous material itself. Sesame and moringa oils are not commonly used in conservation practices. Therefore, while deamidation results for the Nebamun wall painting proteins are not compatible with ancient/original plant products, a more modern origin of the material is unlikely.

With regard to the cereal proteins, none of the samples - green and glue paint from EA37985, and blue paint from EA 921 - showed any degree of deamidation (Fig. S17), which is likely due to the insufficient number of peptides that can undergo deamidation. Discussion on the degradation level of these materials based on deamidation is therefore inconclusive.

In addition to deamidation, a mass-shift search for other post-translational modifications (PTMs) using the Dependent Peptides algorithm of MaxQuant, and a more specific search addressed to identify protein damage related to photo-oxidation, following the known variable modifications proposed by Mackie et al. (27), were performed. With regard to the plant proteins,

and in particular to the sesame and moringa seed storage proteins, we did a search for heat-induced PTMs (89) to possibly gain more information on the seeds treatment. None of these investigations led to any notable result.

Wild animals in ancient Egypt

Among the Alcelaphinae and Cephalophinae subfamilies, the species *Damaliscus lunatus* (sassaby) together with *Connochaetes taurinus* (blue wildebeest), and *Philantomba maxwellii* (Maxwell's duiker), were the only plausible species, among a small range (Table S2), consistent with the geographical origin of the artworks, that were identified (MM 13944 sample S7, EA 6666 sample S2, ÆIN 1522 sample S2, ÆIN 641 sample S1). The species *Oryx gazella* (gemsbok) and *Oryx dammah* (scimitar oryx) were instead identified within the *Oryx* genus. Among these African antelopes, *O. dammah* is the only one native to Egypt (90). Representations of antelope slaughter were discovered in Old and Middle kingdom tombs (3000 - 1600 BCE ca.), and faunal remains of wild animals, including *O. dammah* and other unidentified antelopes, were confirmed in the Fayum area since the Neolithic period (6000 BCE ca.) (91).

Chicken in ancient Egypt

With regard to the identification of the species *Gallus gallus*, according to the literature, domestic chicken (*G. g. domesticus*) was not common in Egypt during the Dynastic period, which corresponds to the date of the object under study, and no faunal record is known prior to the Ptolemaic period (332 – 30 BCE) (92). However, it should also be noted that eggs from wild ancestors of domesticated chicken such as the red jungle fowl and its subspecies (*Gallus gallus* subsp.) would show similar protein sequences, and still be consistent with the archeological findings (93). The artistic use of egg in different artworks from ancient Egypt has been previously suggested in a few studies by using GC-MS and enzyme-linked immunosorbent assay (ELISA) which showed the use of egg as both paint binder (18, 2) and as coating (4). This later application of specifically chicken whole egg and egg white was also confirmed by proteomics in a more recent study of ancient Egyptian funerary portraits (1st century BCE - 2nd century CE) (34).

Sesame, moringa and cereals in ancient Egypt

The date of introduction of sesame into Egypt is still under debate: some interpretations of texts suggest that sesame was cultivated during the Ptolemaic period (332 – 30 BCE) (52), but archaeobotanical evidence seems to suggest an earlier introduction of the plant in the New Kingdom (possibly in the later 14th century BCE) (53). With regard to the moringa tree, both the tree and its oil are mentioned in Egyptian texts from 2635 BCE (7). The first archaeobotanical remains of moringa nuts date to the Graeco-Roman period (332 BCE - 395 CE) (53), which would support the identification of this plant in the wooden coffin investigated in this study (MM 13944), dated from the same period. The archaeobotanical findings specifically refer to the native African species *M. peregrina*, since there is no evidence that the species *M. oleifera*, native to the Indian subcontinent, was known in Egypt at that time (94). The protein attribution in our study to the species *M. oleifera*, and not *M. peregrina*, can be explained by the fact that

albumins have not been sequenced for *M. peregrina* yet or are not publicly available. Therefore, due to sequence similarity, we cannot exclude that the seed proteins originate instead from *M. peregrina*, or even another *Moringa* species. Proteomics data is shared publicly and can be used as public databases expand (See 'Data and materials availability' in the main manuscript).

Emmer wheat (*Triticum dicoccoides*) was discovered in jars in Tutankhamun's tomb (94) and it was also found, together with barley (*Hordeum vulgare*), in three different settlement sites covering the periods from the Old Kingdom (ca 2700 - 2200 BCE) to the 3rd Intermediate Period (1070 - 664 BCE) (95). The identification of barley proteins in the wall painting EA 921, dated 1425-1400 BCE, is therefore consistent with the archaeobotanical records, while the presence in the same painting of proteins from *Triticum* species other than *T. dicoccoides*, requires a more critical interpretation. *T. dicoccoides* was the most common crop until the Graeco-Roman period (332 BCE - 395 CE), when emmer wheat was substituted with bread wheat (*Triticum aestivum*) (96, 97). The species *T. aestivum*, identified in the three samples, was introduced around 1000 years after the date of both wall paintings (95-97). However, this species is overrepresented in the publicly available databases with 484,698 proteins compared to the 88,613 for *T. dicoccoides* (NCBI) and 260,429 versus 708 (Uniprot) (accessed February 2026). It is therefore possible that the identified proteins from *T. aestivum* might come from a more ancient *Triticum* crop, currently without publicly available sequences. Moreover, several peptides could be uniquely assigned to the species *Triticum urartu*, a wild diploid grass species related to wheat. This grass is geographically confined to the Middle East (fertile crescent belt) (97); therefore, it should not be considered plausible for the geographical area of interest. However, the identification of proteins from this species could be explained by the fact that *T. urartu* contributed chromosomes to the tetraploid *T. turgidum* and the hexaploid *T. aestivum*, both identified in the samples.

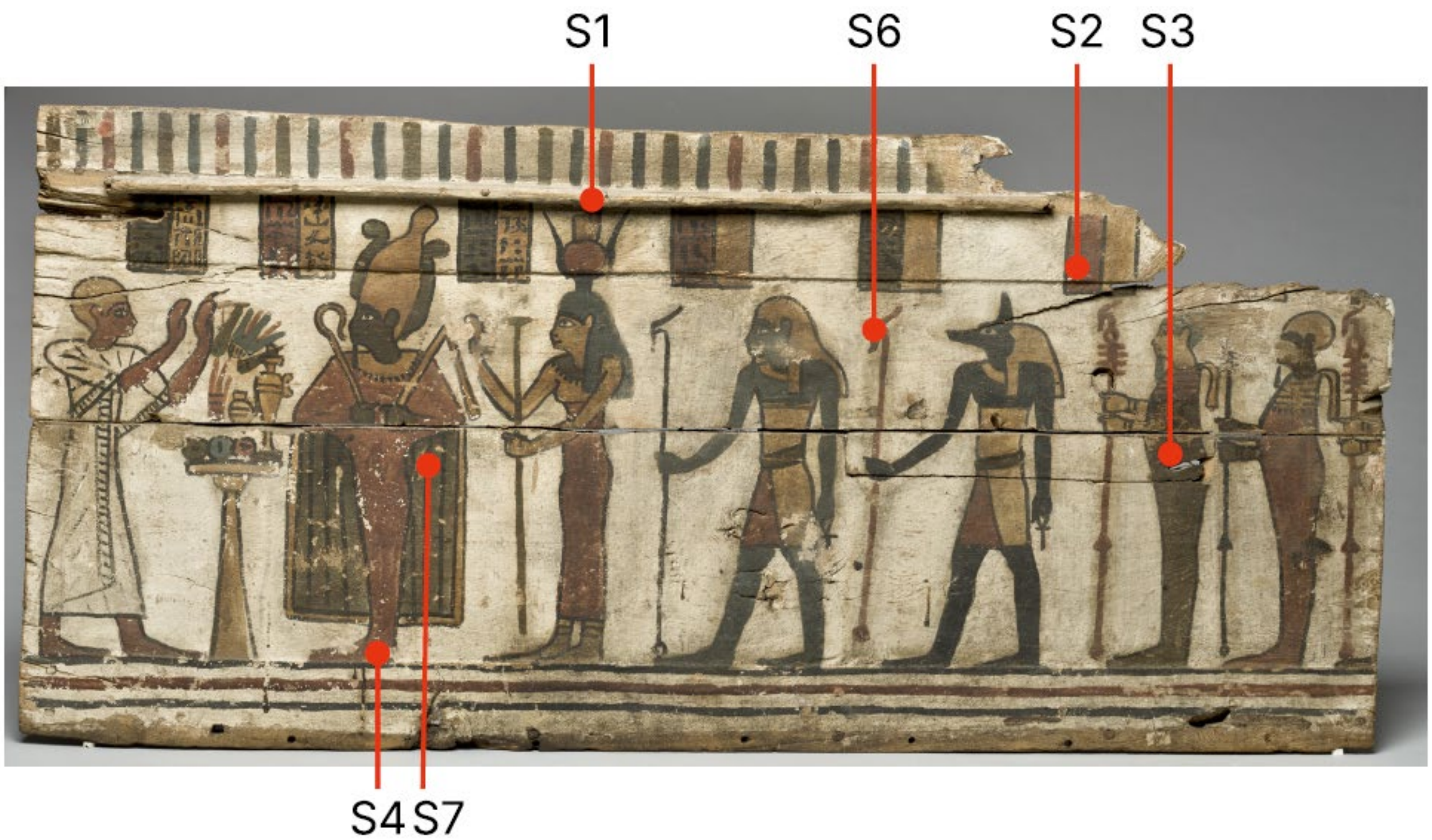

Fig. S1. Fragment of wooden coffin.
Fragment of a painted wooden mummy coffin (Medelhavsmuseet, MM 13944), painted wood, H. 47.5 cm, W. 145 cm, showing the sampling spots. Photo credit: Images courtesy of the Museum of Mediterranean and Near Eastern Antiquities, Stockholm.

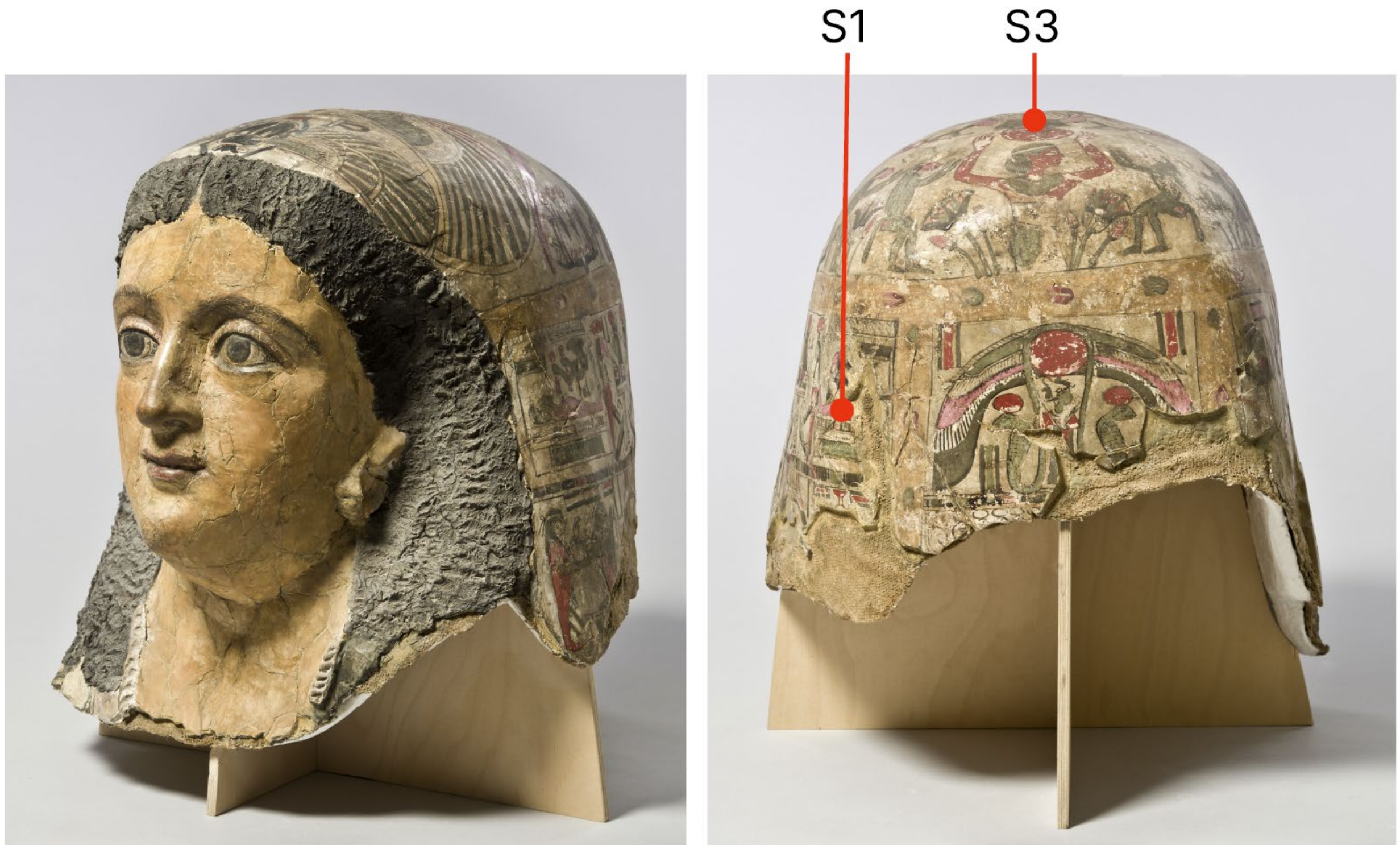

Fig. S2. Mummy mask.
Mummy mask of a woman (Medelhavsmuseet, MM 14957), stucco on linen, H. 37 cm, W. 33 cm, D. 38 cm, showing the sampling spots. Photo credit: Images courtesy of the Museum of Mediterranean and Near Eastern Antiquities, Stockholm.

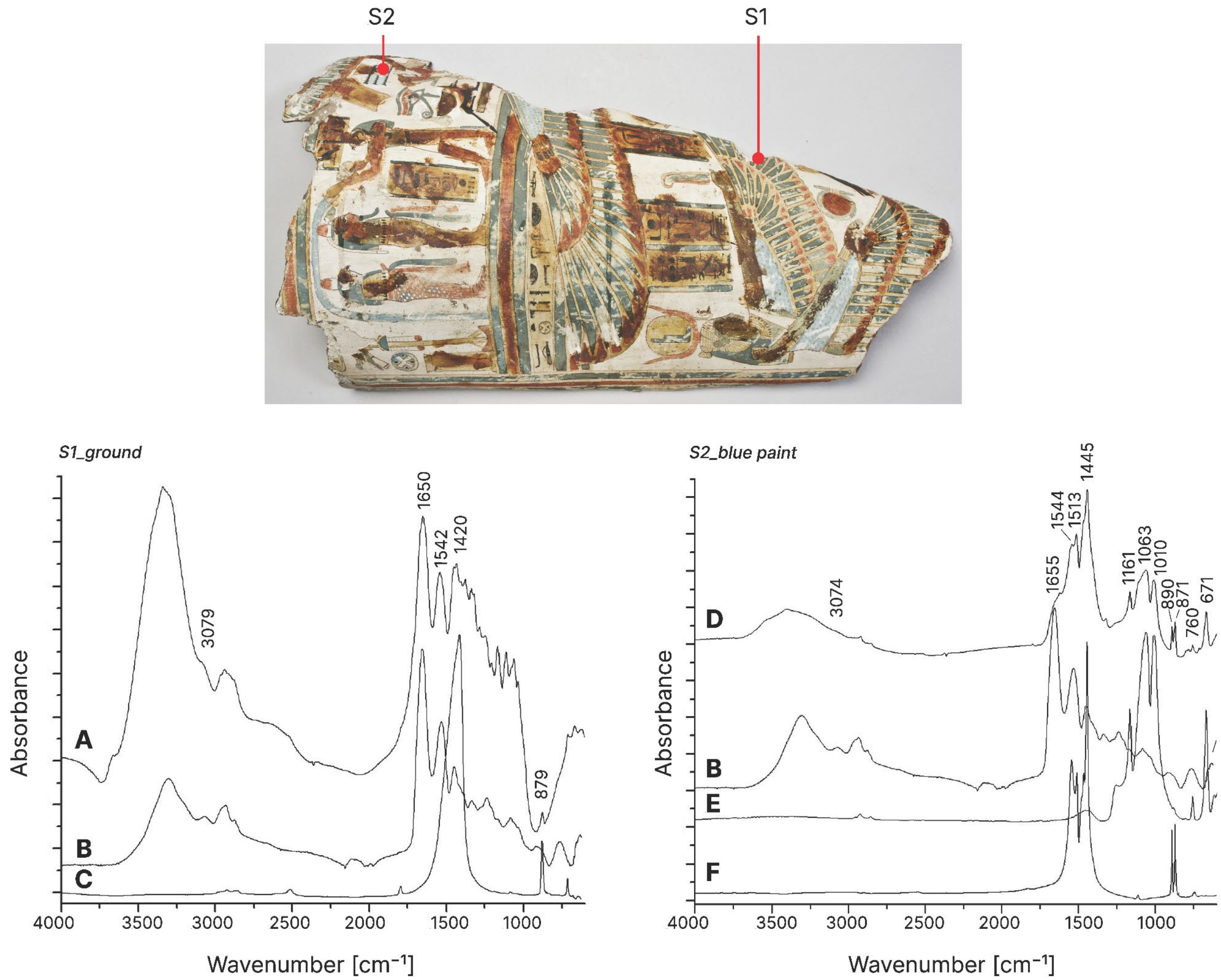

Fig. S3. Fragment of cartonnage.

Top: Fragment of a painted mummy cartonnage (Medelhavsmuseet, MM 18156), L. 59 cm, showing the sampling spots. Photo credit: Images courtesy of the Museum of Mediterranean and Near Eastern Antiquities, Stockholm. Bottom: (left) FTIR spectra of (**A**) ground sample S1, (**B**) reference animal glue (IRUG database PR00017) (98), (**C**) reference calcium carbonate (IRUG MP00241); (right) FTIR spectra of (**D**) blue paint sample S2, (**E**) reference Egyptian blue (IRUG MP00005), and (**F**) reference huntite (IRUG MP00386).

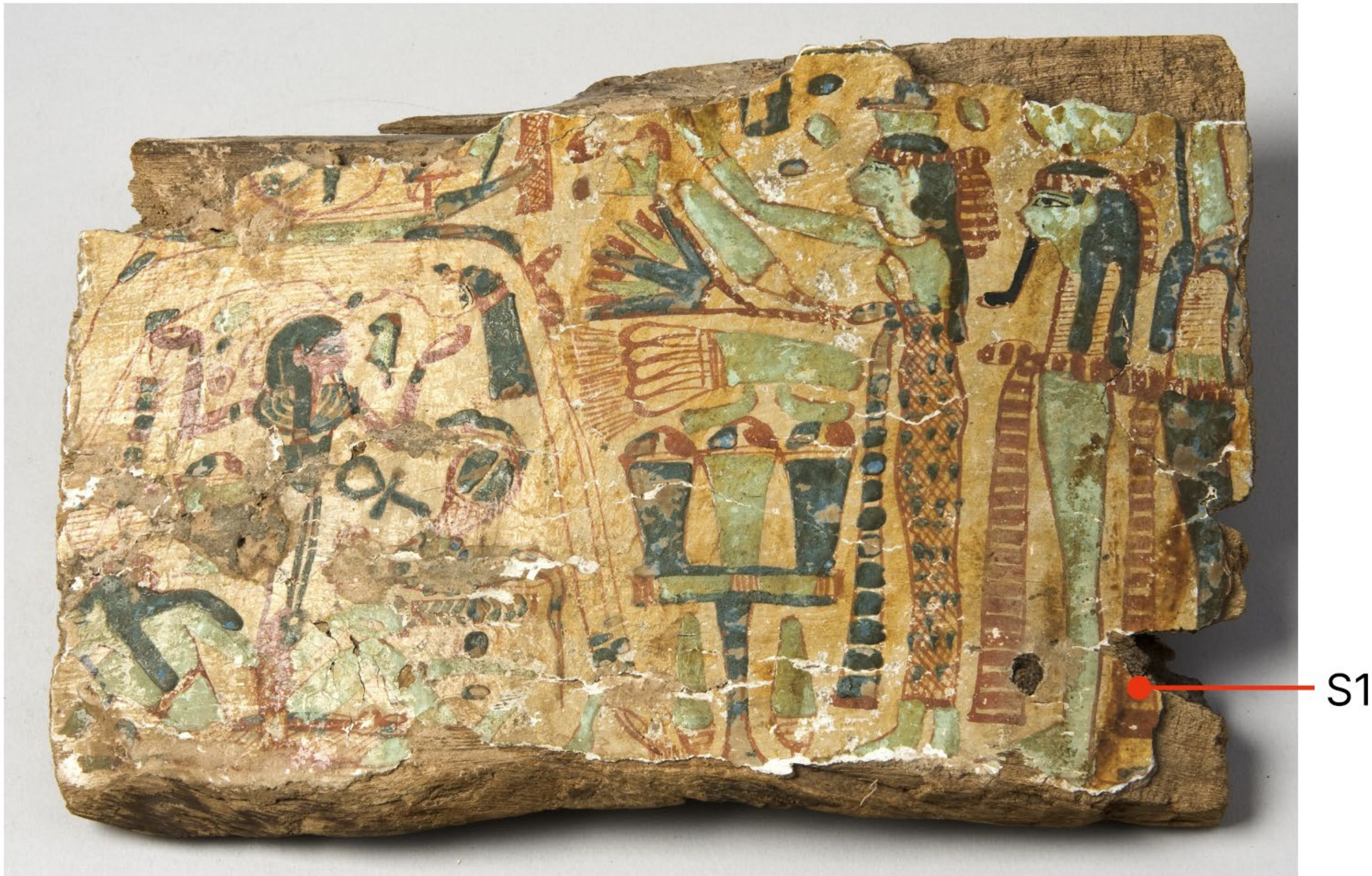

Fig. S4. Fragment of wooden coffin.
Fragment of a painted wooden mummy coffin (Medelhavsmuseet, MME 1977:018), H. 21 cm, L. 29 cm, showing the sampling spot. Photo credit: Images courtesy of the Museum of Mediterranean and Near Eastern Antiquities, Stockholm.

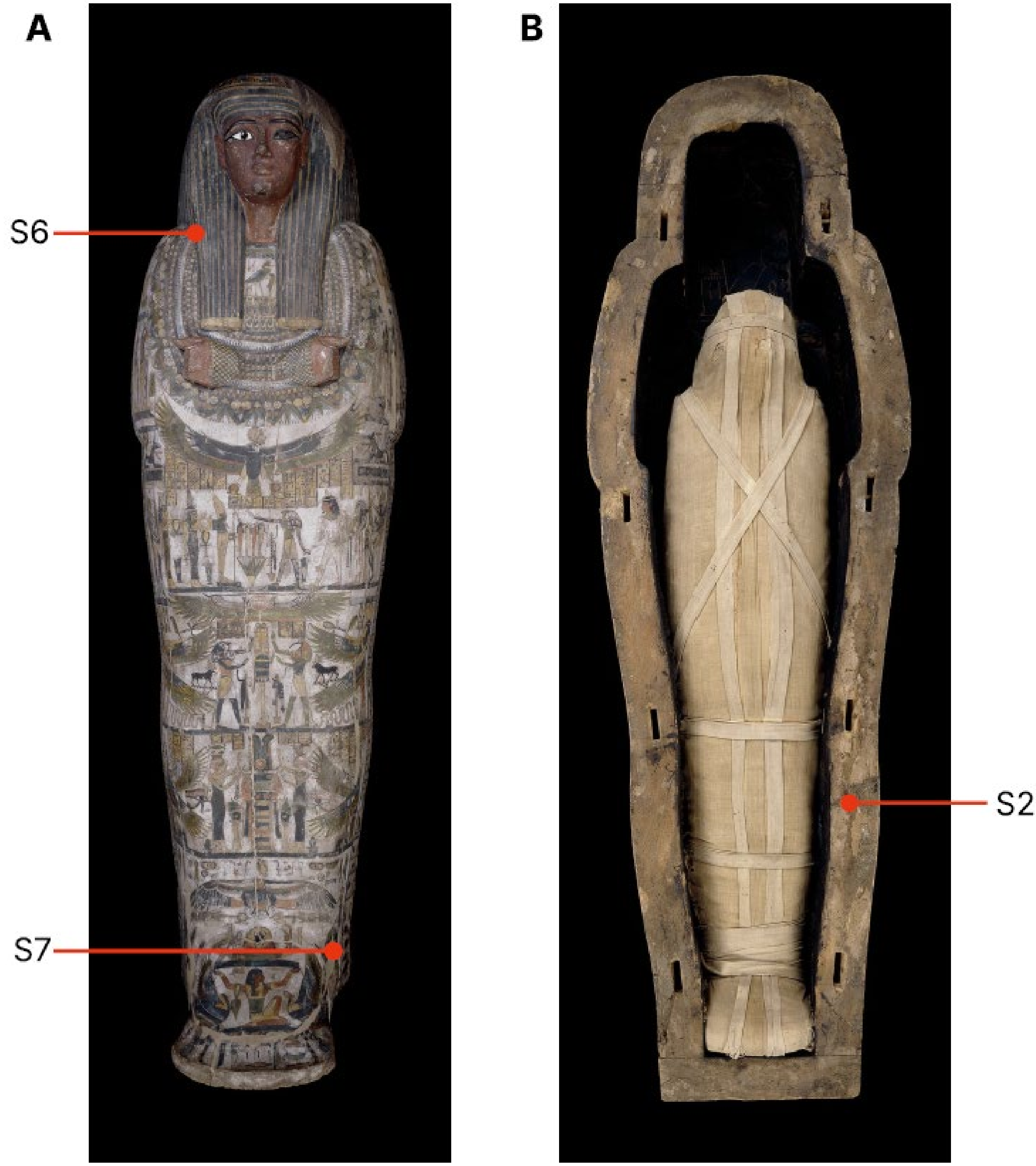

Fig. S5. Wooden coffin adhesive.
Painted wooden coffin of Horaawesheb, from Abydos (British Museum, EA 6666) L. 155 cm, showing the sampling spots: (A) lid, and (B) case. © The Trustees of the British Museum. Photo Credit: shared under a Creative Commons Attribution-NonCommercial-ShareAlike 4.0 International (CC BY-NC-SA 4.0) licence, British Museum.

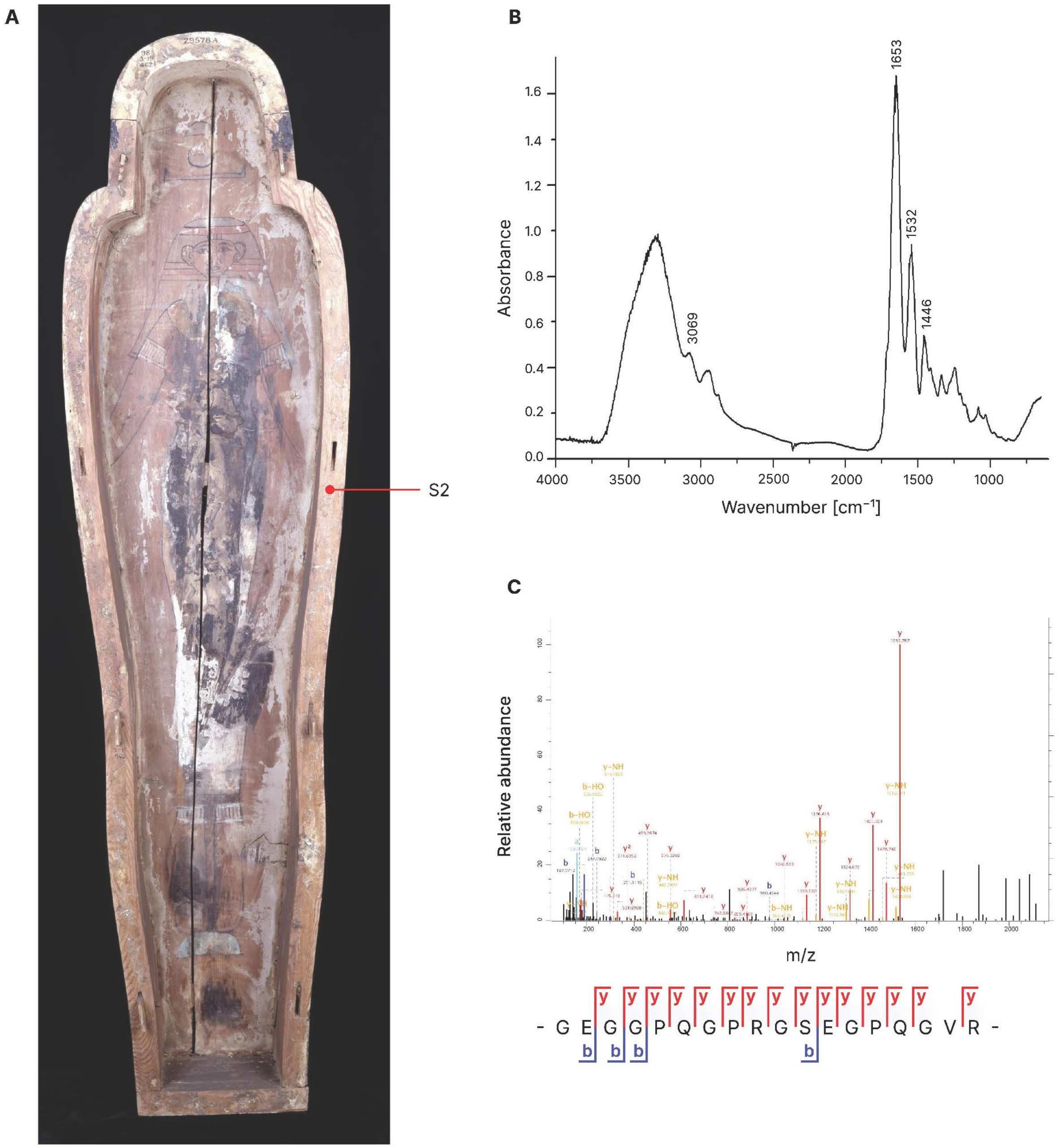

Fig. S6. Wooden coffin adhesive, FTIR and MS/MS spectra.
(**A**) Painted wooden coffin of Padihorpakhered, Thebes (British Museum, EA 29578) L. 167 cm, showing the sampling spot. © The Trustees of the British Museum. Photo Credit: shared under a Creative Commons Attribution-NonCommercial-ShareAlike 4.0 International (CC BY-NC-SA 4.0) licence, British Museum. (**B**) FTIR spectrum of sample S2 (adhesive) with bands characteristic of protein-based materials; (**C**) MS/MS spectrum of a peptide from collagen 1 alpha 1 from *Bos taurus*.

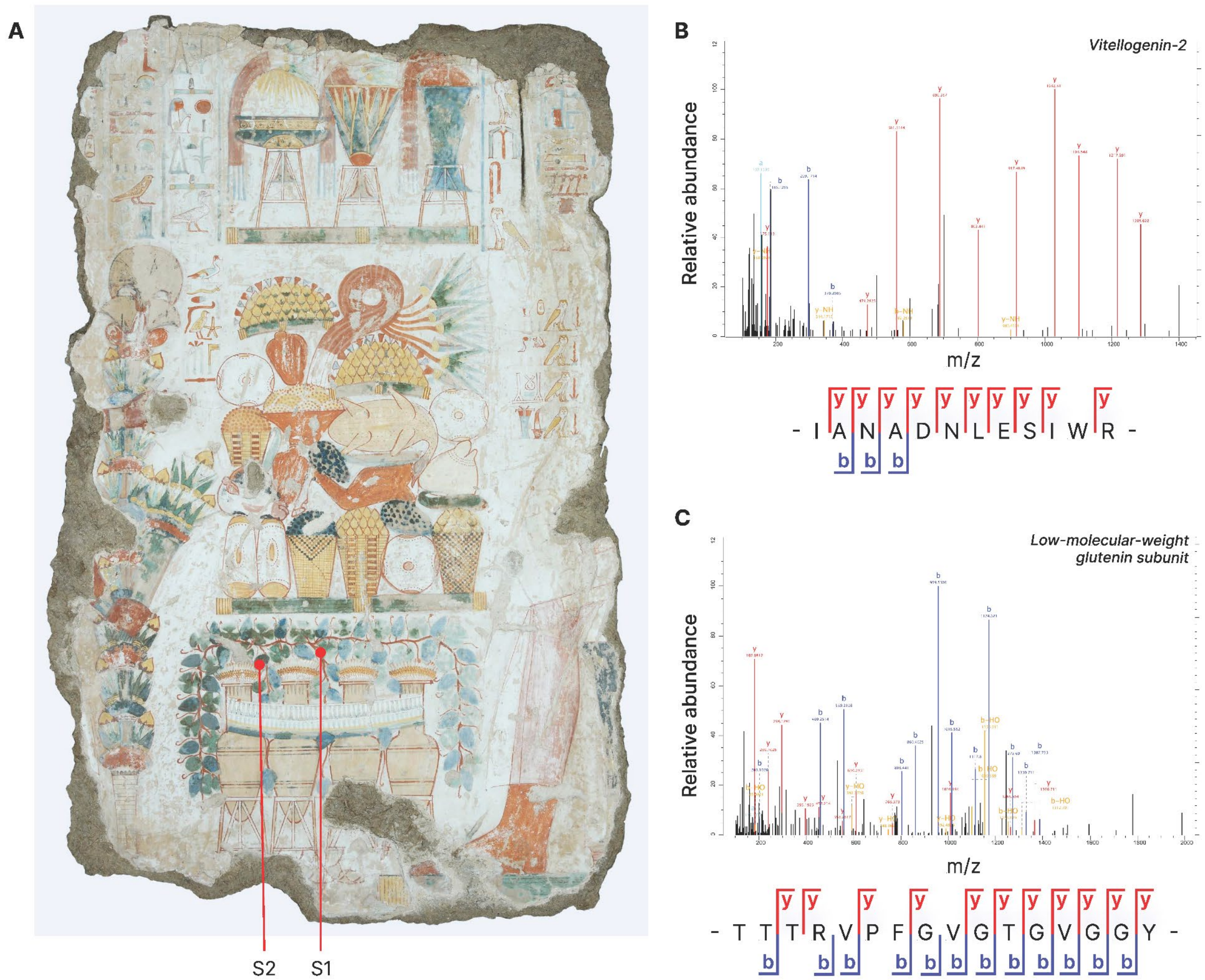

Fig. S7. Wall painting.
(A) Wall painting fragment from the tomb of Nebamun, Thebes (British Museum, EA 37985), painted plaster, L. 104.2, W. 61 cm, showing the sampling spots. © The Trustees of the British Museum. Photo Credit: shared under a Creative Commons Attribution-NonCommercial-ShareAlike 4.0 International (CC BY-NC-SA 4.0) licence, British Museum. MS/MS spectrum of **(B)** a peptide from Vitellogenin-2 from *Gallus gallus*, and **(C)** a peptide from Low-molecular-weight glutenin subunit from the genus *Triticum* in green paint sample S1.

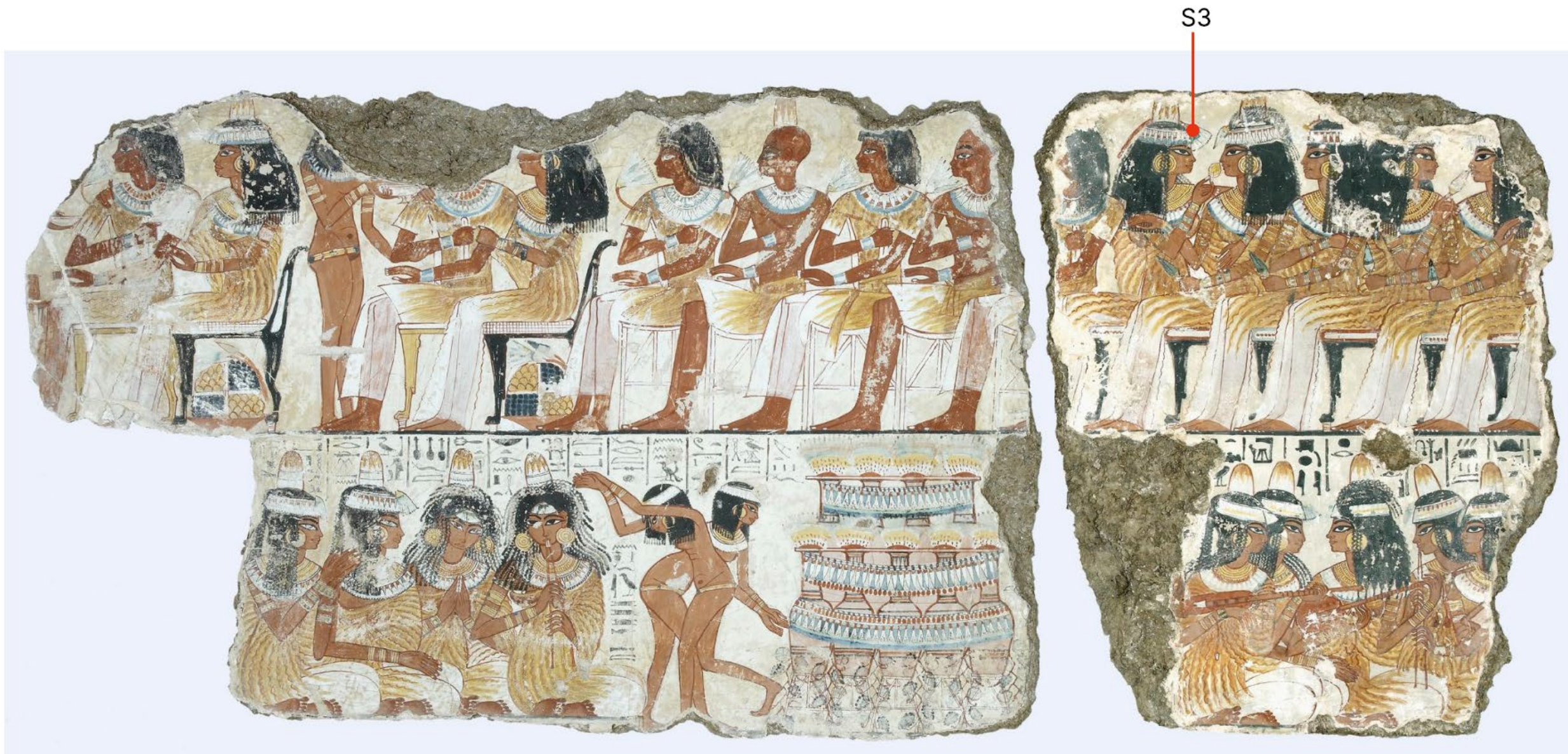

Fig. S8. Wall painting.
Wall painting fragment from the tomb of Nebamun, Thebes (British Museum, EA 37981), painted plaster, W. 50 cm, showing the sampling spot. © The Trustees of the British Museum. Photo Credit: shared under a Creative Commons Attribution-NonCommercial-ShareAlike 4.0 International (CC BY-NC-SA 4.0) licence, British Museum.

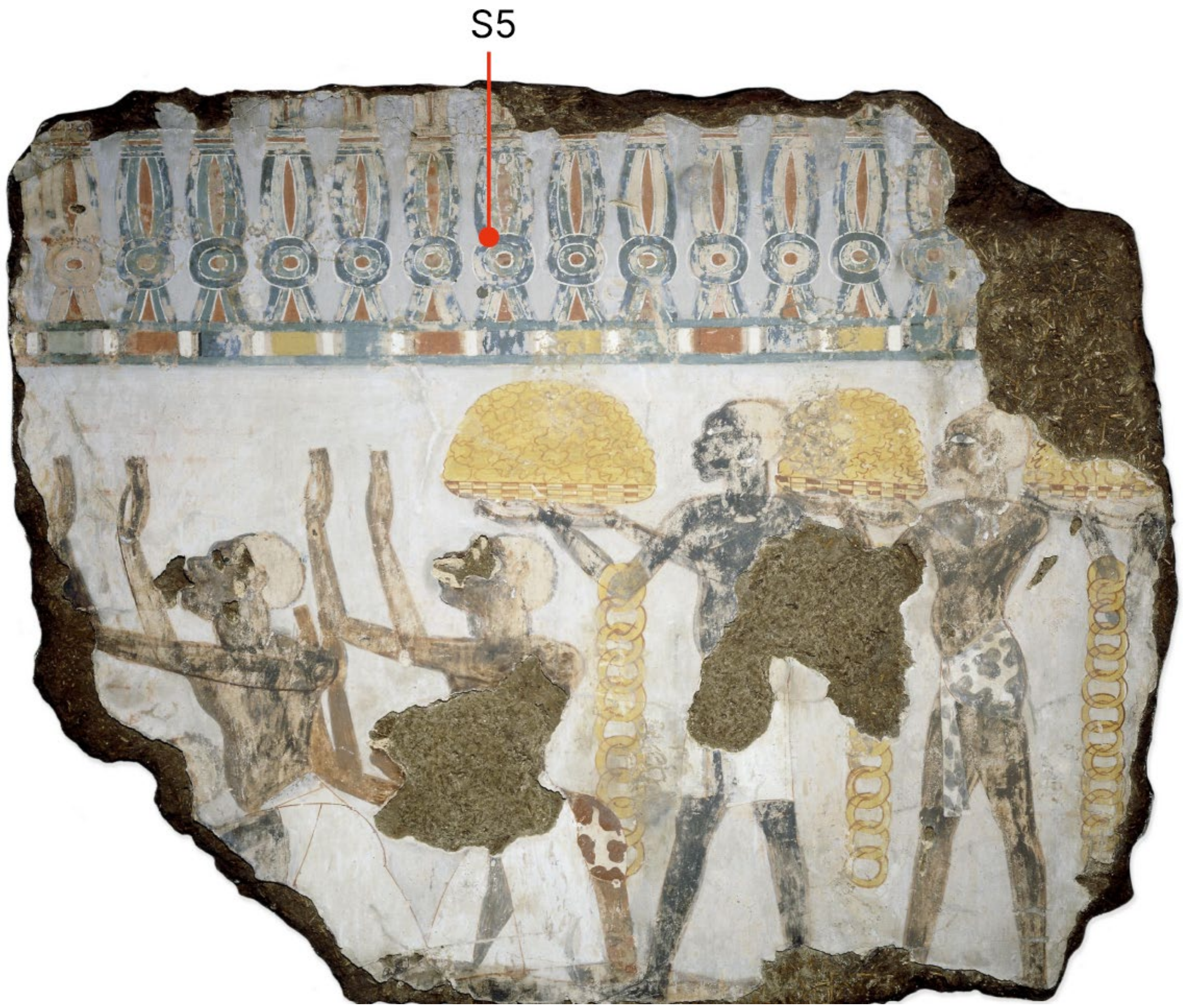

Fig. S9. Wall painting.
Fragment of a tomb wall from the Tomb of Sobekhotep (British Museum, EA 921), H. 71 cm, W. 96.5 cm, plaster on mud, showing the sampling spot. © The Trustees of the British Museum. Photo Credit: shared under a Creative Commons Attribution-NonCommercial-ShareAlike 4.0 International (CC BY-NC-SA 4.0) licence, British Museum.

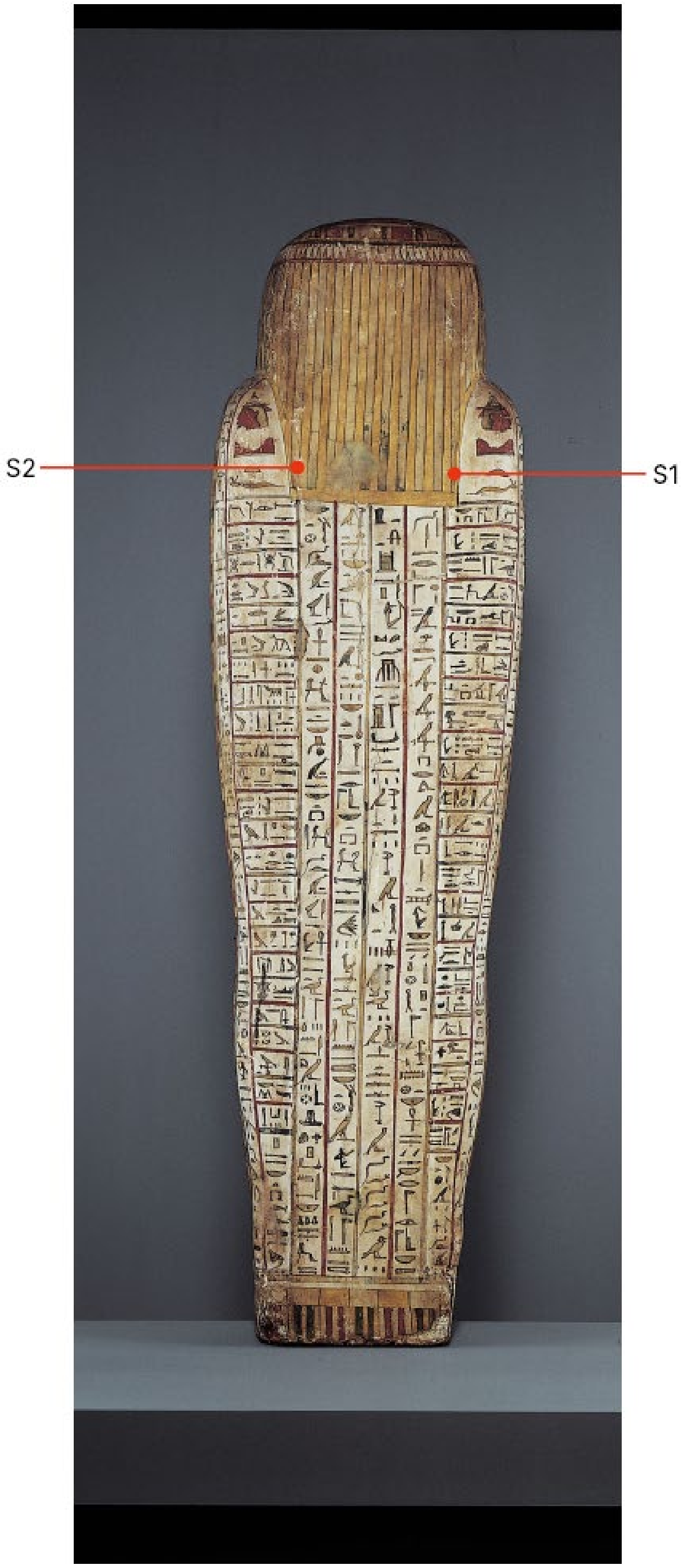

Fig. S10. Wooden coffin.
Painted wooden coffin of Gaut-seshen from Deir el-Bahrie, back (Ny Carlsberg Glyptotek, ÆIN 1522), L. 183 cm, showing the sampling spots. Photo credit: Ole Haupt, Ny Carlsberg Glyptotek.

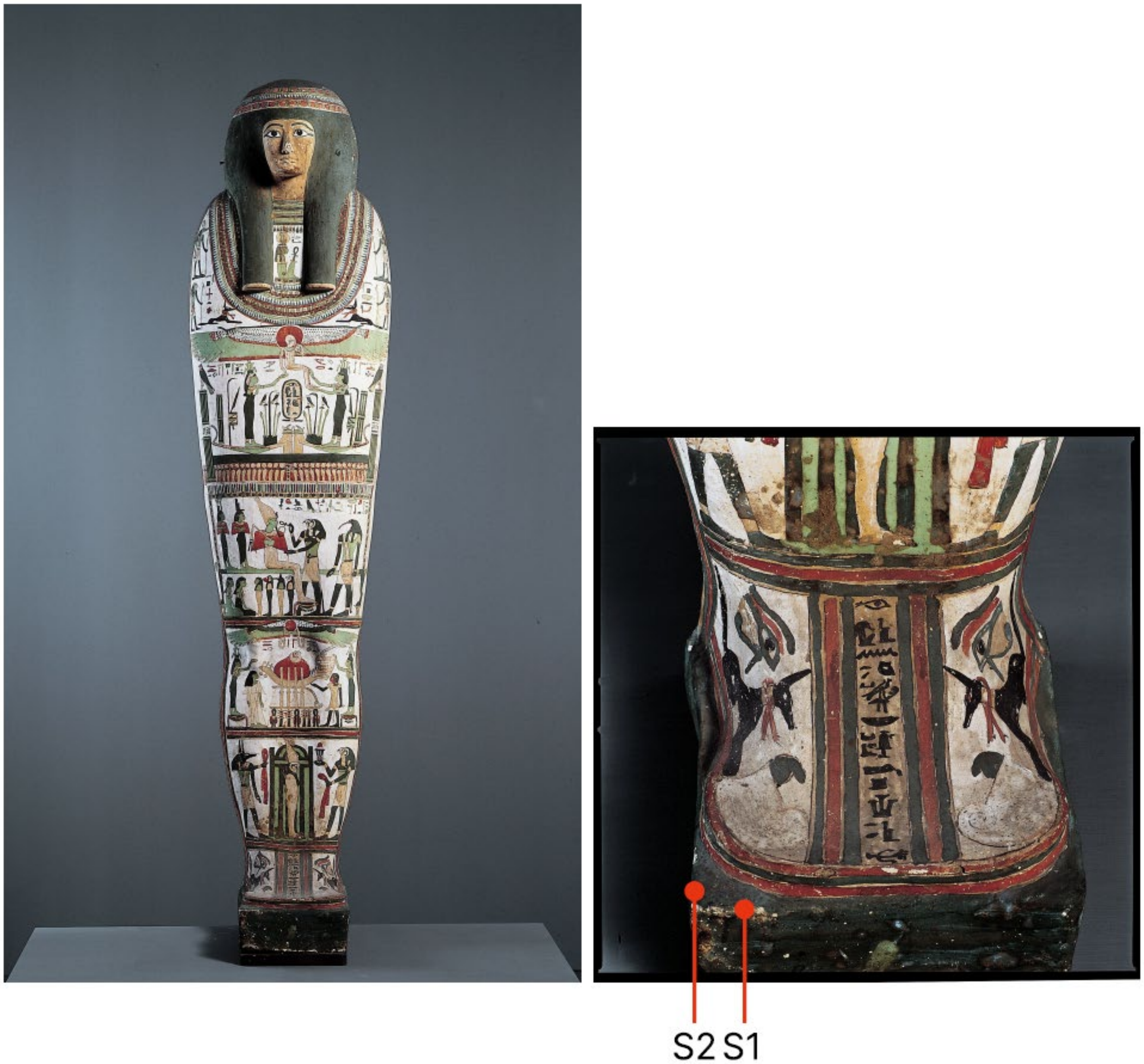

Fig. S11. Wooden coffin.
Painted wooden coffin of Kep-ha-ese (Ny Carlsberg Glyptotek, ÆIN 298), L. 160 cm (left) and close up of the bottom part of the coffin showing the sampling spots (right). Photo credit: Ole Haupt, Ny Carlsberg Glyptotek.

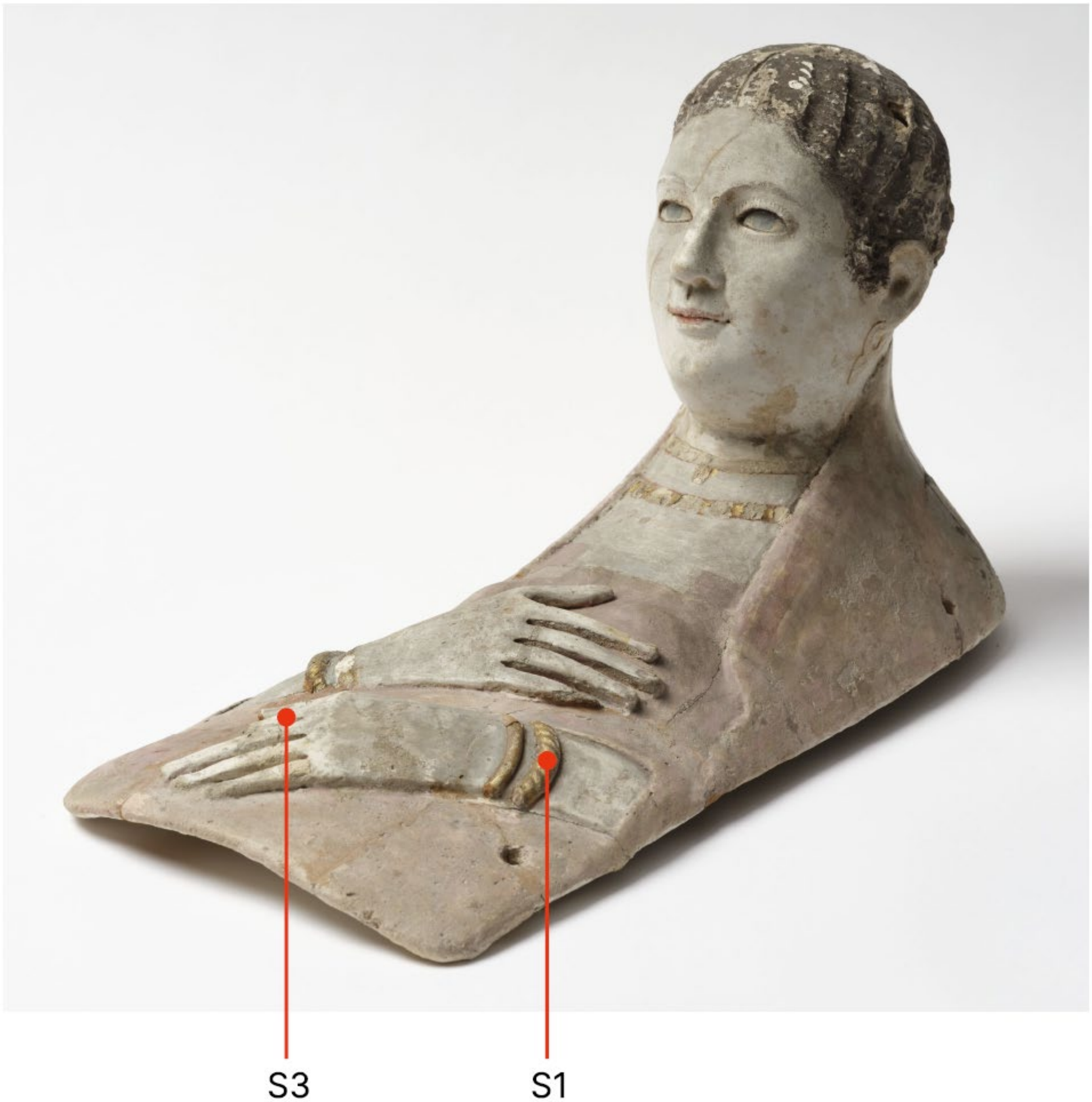

Fig. S12. Mummy bust.
Mummy bust (Ny Carlsberg Glyptotek, ÆIN 641), painted plaster, gilding, and glass (eyes), H. 32 cm, L. 53 cm, showing the sampling spots. Photo credit: Anders Sune Berg, Ny Carlsberg Glyptotek.

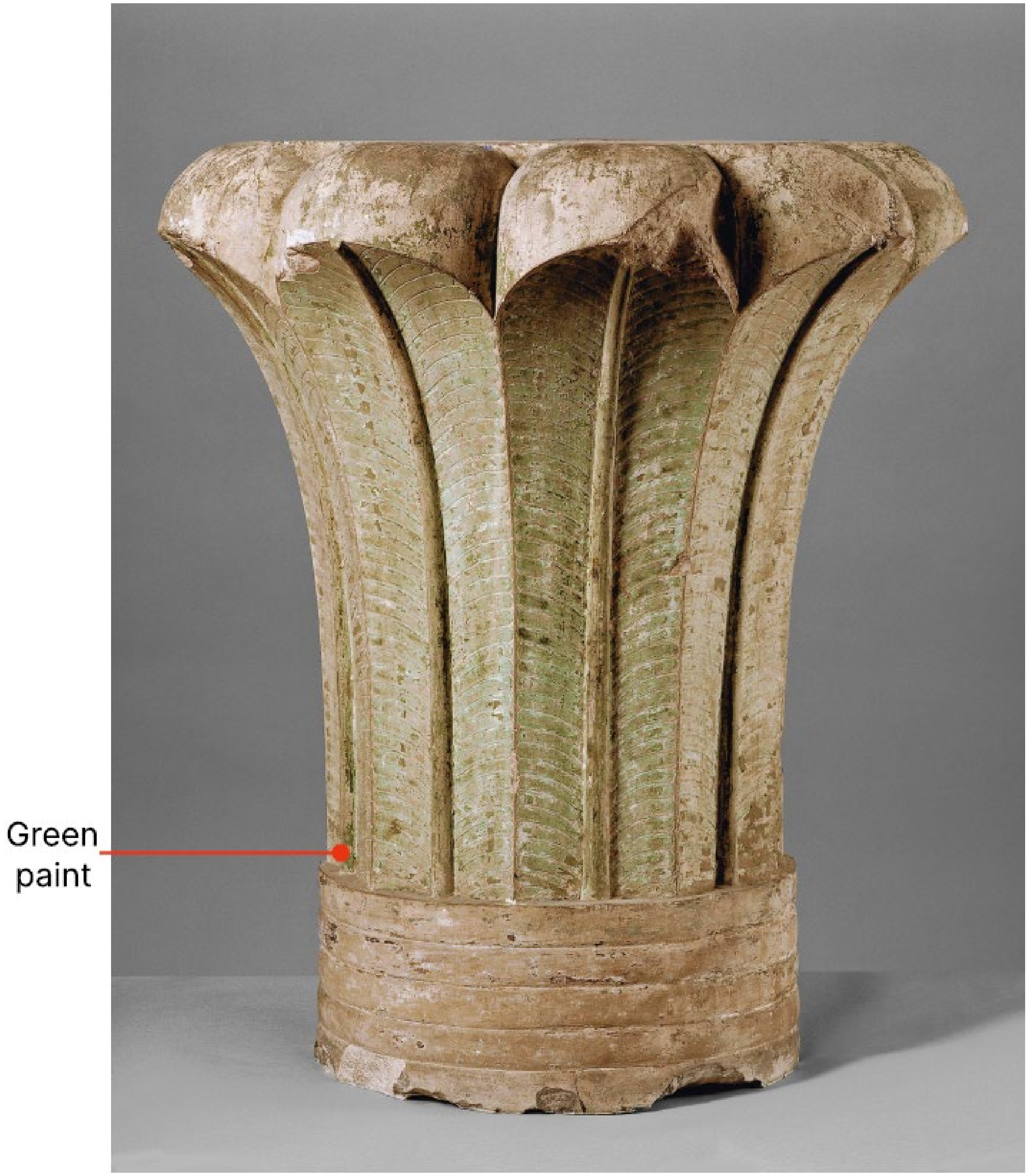

Fig. S13. Column capital.
Column capital from the palace of Apries, Memphis (Ny Carlsberg Glyptotek, ÆIN 1045), painted limestone, H. 85 cm, D. 75/44 cm, showing the sampling spot. Photo credit: Ole Haupt, Ny Carlsberg Glyptotek.

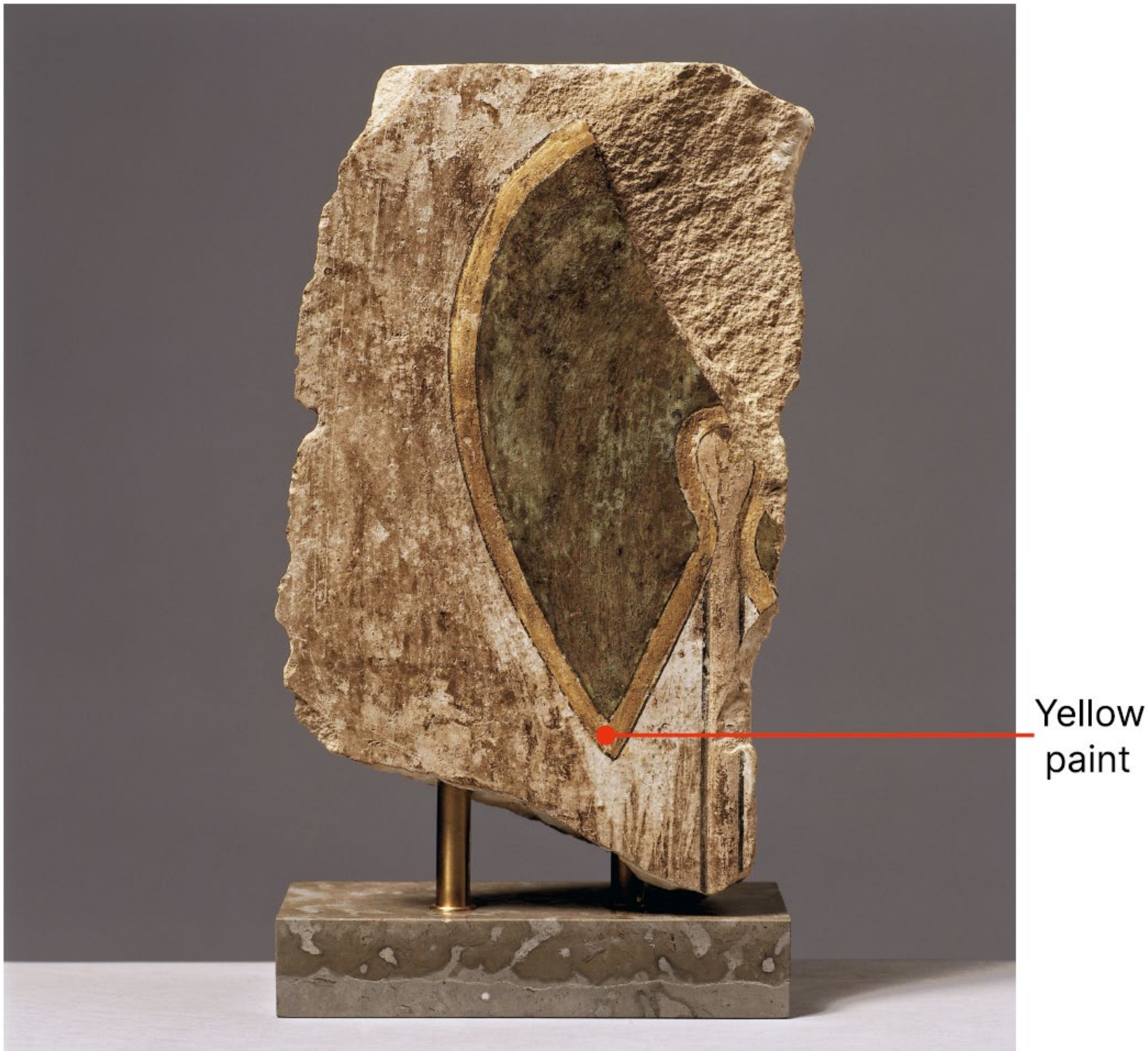

Fig. S14. Architectural fragment.
Relief fragment from the palace of Apries, Memphis (Ny Carlsberg Glyptotek, ÆIN 1060), painted limestone, H. 24.3, W. 14.6, showing the sampling spot. The sample named ‘white surface layer’ consists of a scraping from the surface of the yellow paint sample. Photo credit: Ole Haupt, Ny Carlsberg Glyptotek.

Table S1. Proteins identified.
Proteins identified in the samples analysed: object accession number, sample number, sample description, protein accession number when available, protein name, taxonomy source, number of razor + unique peptides supporting the identification, percentage of total and unique sequence coverage, sequence length and total number of identified MS/MS spectra.

Table S2. Peptides identified.
Taxonomic specific peptides identified in the samples analysed: object accession number, sample number, sample description, protein name and accession number when available, peptide sequence, species matching on the NCBI database, peptide length and mass, MaxQuant score, and total number of identified MS/MS spectra. Species in parentheses are unlikely due to geographical or historical reasons.

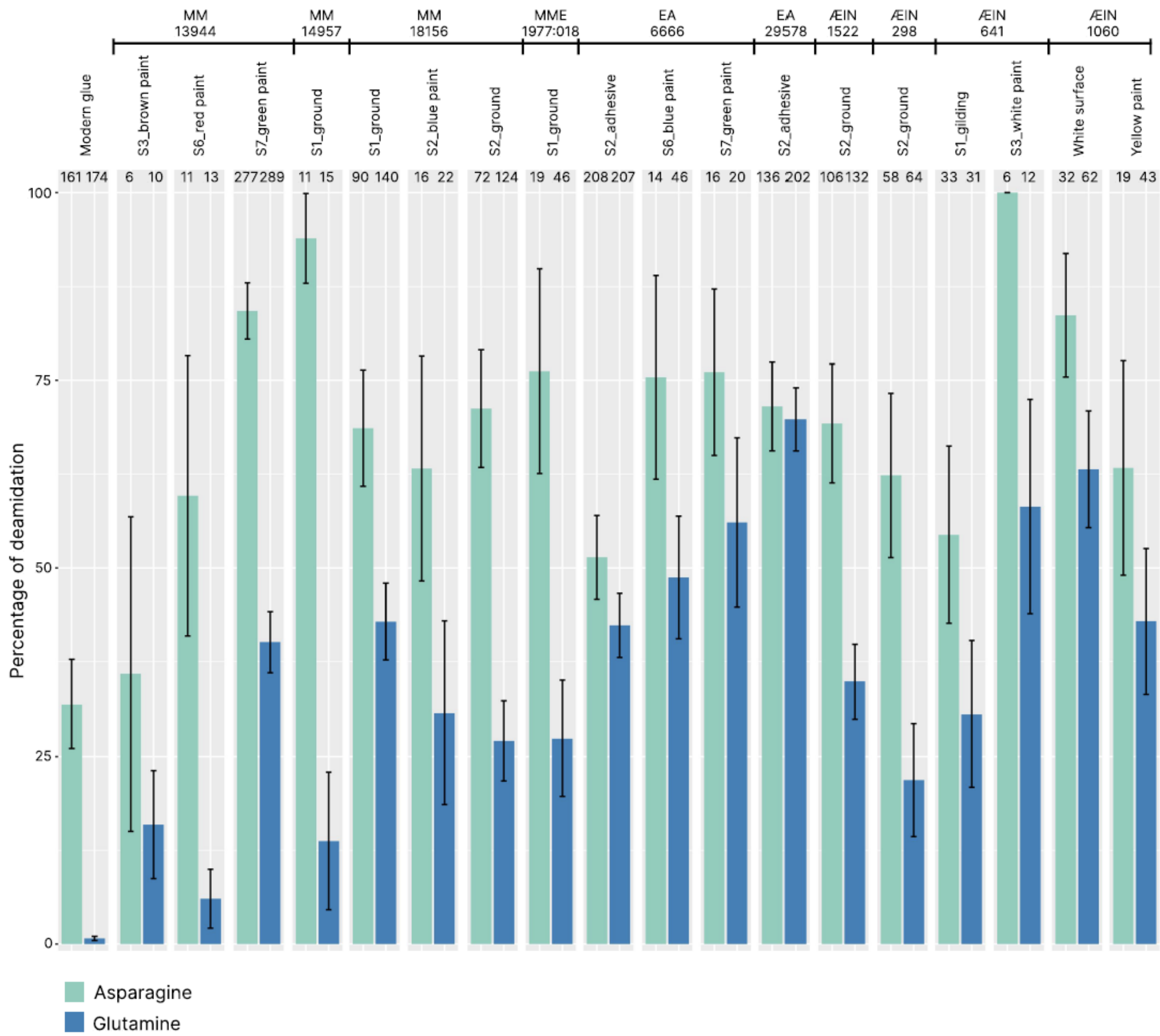

Fig. S15. Deamidation level of collagen proteins.
Deamidation rate (%) of Asn (N) and Gln (Q) for the collagen proteins, regardless of the animal species, identified in single paint or ground layers of historic samples and in a modern glue (PRIDE dataset identifier PXD024397, raw file “E_large_1299.raw”) (30). Error bars represent the standard deviation and the numbers on top of the bars indicate the number of peptides used to calculate deamidation.

Sample	N/Q	Number peptides	Mean	STD
ÆIN 1060, coating	N<br>Q	32<br>62	84.1<br>63.7	8.0<br>7.4
ÆIN 1060, yellow paint	N<br>Q	19<br>43	62.7<br>42.8	14.7<br>9.4
MM 18156, S1, ground layer	N<br>Q	90<br>140	68.3<br>42.7	7.9<br>5.2
MM 18156, S2, blue paint	N<br>Q	16<br>22	63.9<br>30.4	14.5<br>13.1
MM 18156, S2, ground layer	N<br>Q	72<br>124	71.1<br>26.9	7.5<br>5.3
EA 29578, adhesive	N<br>Q	136<br>202	71.7<br>69.3	6.2<br>4.2
EA 6666, adhesive	N<br>Q	226<br>241	55.5<br>41.1	5.2<br>4.1
EA 6666, S6, blue paint	N<br>Q	14<br>46	76.6<br>48.8	12.6<br>8.8
EA 6666, S7, green paint	N<br>Q	16<br>20	76.2<br>55.8	11.3<br>11.1
MM 14957, S1, ground layer	N<br>Q	11<br>15	93.9<br>13.9	5.9<br>9.0
MME 1977:018, S1, ground layer	N<br>Q	19<br>46	75.7<br>27.7	13.6<br>7.6
MM 13944, S3, brown paint	N<br>Q	6<br>10	36.5<br>15.9	21.0<br>7.5
MM 13944, S6, red paint	N<br>Q	11<br>13	59.3<br>6.0	19.1<br>4.1
MM 13944, S7, green paint	N<br>Q	347<br>374	83.5<br>38.8	3.4<br>3.7
ÆIN 1522, S2, ground layer	N<br>Q	164<br>174	65.4<br>34	6.7<br>4.5
ÆIN 298, S2, ground layer	N<br>Q	58<br>64	62.1<br>21.9	10.5<br>7.5
ÆIN 641, S1, gilding	N<br>Q	33<br>31	54.5<br>30.8	12.1<br>9.5
ÆIN 641, S3, white paint	N<br>Q	6<br>12	100.0<br>59.1	0.0<br>14.8
Modern glue	N<br>Q	161<br>174	31.9<br>0.8	5.9<br>0.3

Table S3. Deamidation percentage and standard deviation for collagen proteins. Percentage of deamidation and corresponding standard deviation of asparagine (N) and glutamine (Q) for the collagen proteins identified in historic samples and in the modern glue

(PRIDE dataset identifier PXD024397, raw file "E_large_1299.raw") (30). The number of peptides used to calculate deamidation for each amino acid is also reported.

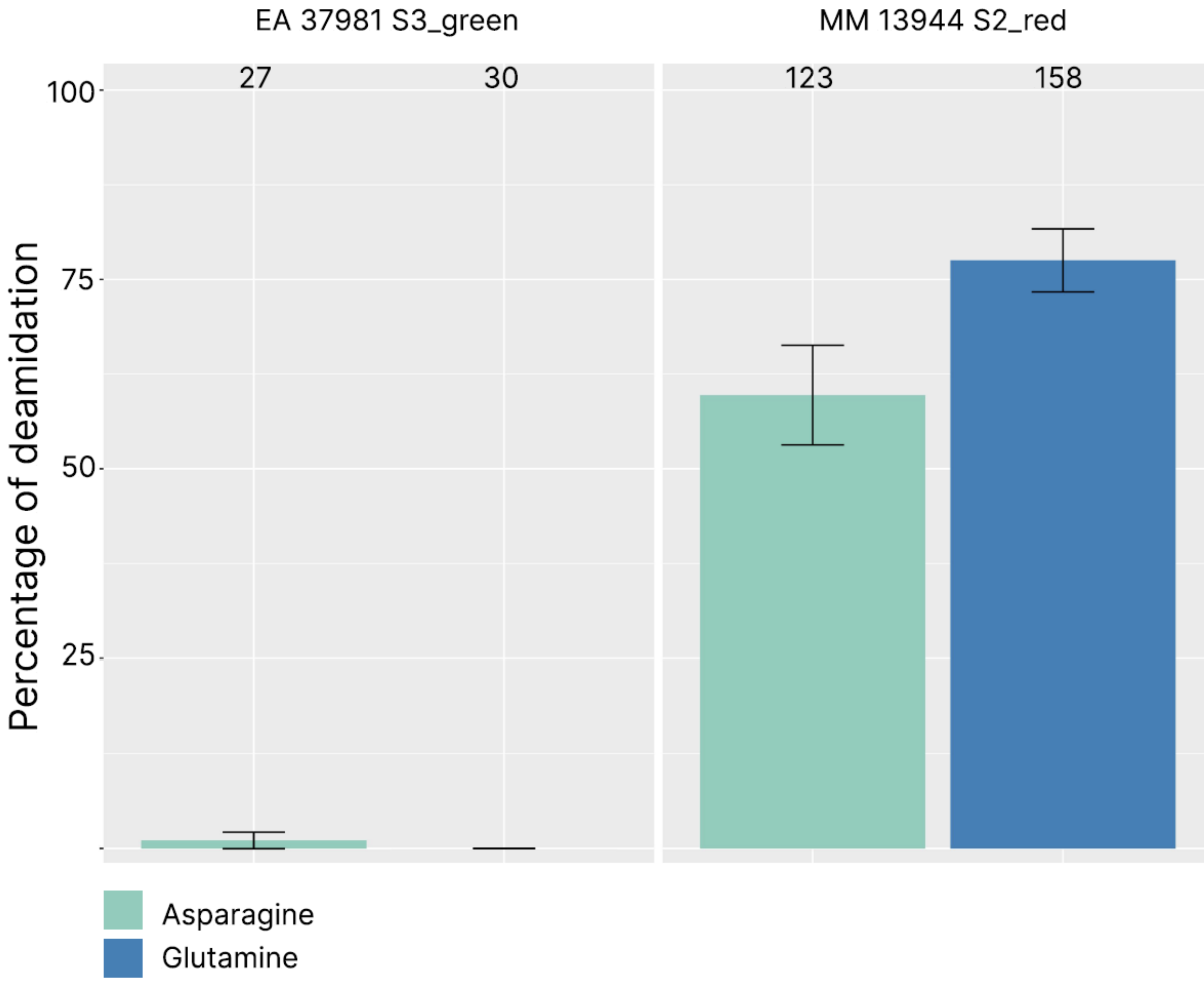

Fig. S16. Deamidation level for seed storage proteins.
Percentage of deamidation of asparagine (N) and glutamine (Q) for the taxonomic specific seed storage proteins identified in the green paint sample (S3) from the tomb of Nebamun wall painting (EA 37981) and in the red paint sample (S2) from a wooden coffin (EA 13944). Error bars represent the standard deviation and the numbers on top of the bars indicate the number of peptides used to calculate deamidation.

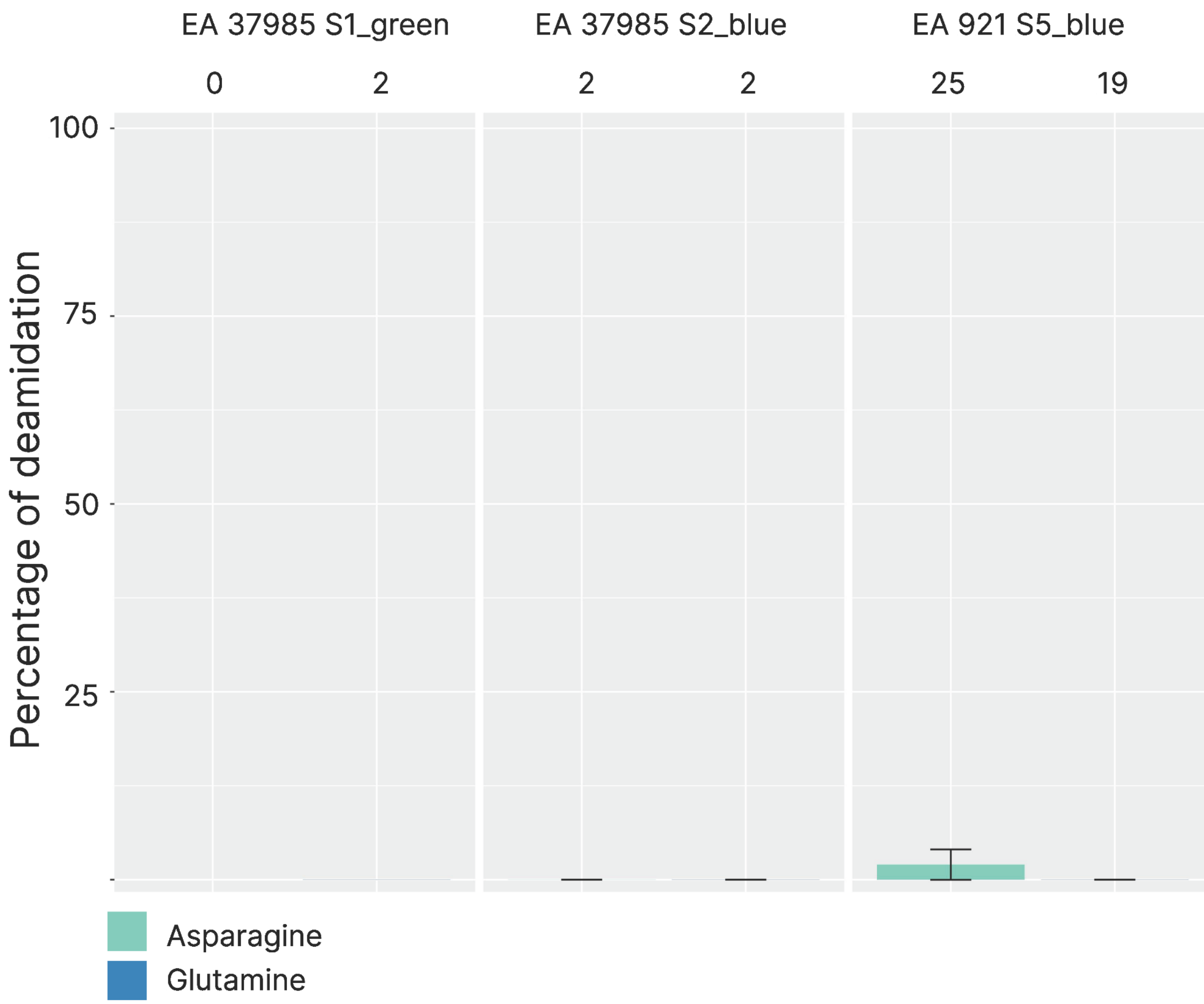

Fig. S17. Deamidation level for cereal proteins.
Percentage of deamidation of asparagine (N) and glutamine (Q) for the taxonomic specific cereal proteins identified in the green (S1) and blue (S2) paint samples from the tomb of Nebamun wall painting (EA 37985) and in the blue paint sample (S5) from Tomb of Sobekhotep wall painting (EA 921). Error bars represent the standard deviation and the numbers on top of the bars indicate the number of peptides used to calculate deamidation.

REFERENCES

1. J. Salvant, J. Williams, M. Ganio, F. Casadio, C. Daher, K. Sutherland, L. Monico, F. Vanmeert, S. De Meyer, K. Janssens, C. Cartwright, M. Walton, A Roman Egyptian painting workshop: Technical investigation of the portraits from Tebtunis, Egypt. *Archaeometry* **60**, 815–833 (2018).

2. P. Dietemann, H. Stege, U. Baumer, "Pigmente und Bindemittel antiker Mumienporträts," in *Inkarnat und Signifikanz: Das menschliche Abbild in der Tafelmalerei von 200 bis 1250 im Mittelmeerraum* (Zentralinstitut für Kunstgeschichte, 2017).

3. K. Sutherland, R. Sabino, F. Pozzi, "Challenges in the characterization and categorization of binding media in mummy portraits," in *Mummy Portraits of Roman Egypt: Emerging Research from the APPEAR Project* (J. Paul Getty Museum, 2020).

4. J. Mazurek, M. Svoboda, M. Schilling, GCMS binding media survey: Beeswax, oil, protein, plant gum and resin in Romano-Egyptian mummy portraits and panel paintings. *Heritage* **2**, 1960–1985 (2019).

5. R. Newman, M. Serpico, "Adhesives and binders," in *Ancient Egyptian Materials and Technology* (Cambridge Univ. Press, 2000).

6. C. Granzotto, A. Aksamija, G. H. Tinnevelt, V. Turkina, K. Sutherland, New insight from MALDI-TOF MS and multivariate data analysis on the botanical origin of polysaccharide-based paint binders in ancient Egypt. *Anal. Methods* **16**, 2959–2971 (2024).

7. A. Lucas, J. R. Harris, *Ancient Egyptian Materials and Industries* (Dover, 1999).

8. A. McDonald, "Animals in Egypt," in *The Oxford Handbook of Animals in Classical Thought and Life*, G. L. Campbell, Ed. (Oxford Univ. Press, 2014).

9. D. Gifford-Gonzalez, O. Hanotte, "Domesticating animals in Africa," in *The Oxford Handbook of African Archaeology*, P. Mitchell, P. J. Lane, Eds. (Oxford Academic, 2013).

10. B. J. Collins, *A History of the Animal World in the Ancient Near East* (Brill, 2001).

11. F. Di Gianvincenzo, C. Krarup Andersen, T. Filtenborg, M. Mackie, M. Ernst, J. Ramos Madrigal, J. V. Olsen, J. Wadum, E. Cappellini, Proteomic identification of beer brewing products in the ground layer of Danish Golden Age paintings. *Sci. Adv.* **9**, eade7686 (2023).

12. A. Alexopoulou-Agoranou, A.-E. Kalliga, U. Kanakari, V. Pashalis, "Pigment analysis and documentation of two funerary portraits which belong to the collection of the Benaki Museum," in *Portraits and Masks: Burial Customs in Roman Egypt*, M. L. Bierbrier, Ed. (British Museum Press, 1997).

13. A. Barth, Infrared spectroscopy of proteins. *Biochim. Biophys. Acta Bioenerg.* **1767**, 1073–1101 (2007).

14. S. Rowe, R. Siddall, R. J. Stacey, "Roman Egyptian gilded cartonnage: Technical study and conservation of a mummy mask from Hawara," in *Decorated Surfaces on Ancient Egyptian Objects. Technology, Deterioration and Conservation* (Archetype Publications, 2010).

15. S. P. Sack, F. C. Tahk, T. A. Petersm, Technical examination of an ancient Egyptian painting on canvas. *Stud. Conserv.* **26**, 15–23 (1981).

16. B. Ramer, The technology, examination and conservation of the Fayum portraits in the Petrie Museum. *Stud. Conserv.* **24**, 1–13 (1979).

17. R. J. Stacey, "Paint Media and Varnishes," in *The Nebamun Wall Paintings: Conservation, Scientific Analysis and Display at the British Museum* (Archetype Publications: in association with the British Museum, 2008).

18. C. Brøns, K. L. Rasmussen, M. M. Di Crescenzo, R. J. Stacey, A. Lluveras-Tenorio, Painting the Palace of Apries I: Ancient binding media and coatings of the reliefs from the Palace of Apries, Lower Egypt. *Herit. Sci.* **6**, 6 (2018).

19. T. F. Mathews, N. E. Muller, *The Dawn of Christian Art in Panel Paintings and Icons* (The J. Paul Getty Museum, 2016).

20. M. P. Colombini, F. Modugno, Characterisation of proteinaceous binders in artistic paintings by chromatographic techniques. *J. Sep. Sci.* **27**, 147–160 (2004).

21. S. Orsini, F. Parlandit, I. Bonaduce, Analytical pyrolysis of proteins in samples from artistic and archaeological objects. *J. Anal. Appl. Pyrolysis* **124**, 643–657 (2017).

22. R. Hynek, S. Kuckova, J. Hradilova, M. Kodicek, Matrix-assisted laser desorption/ionization time-of-flight mass spectrometry as a tool for fast identification of protein binders in color layers of paintings. *Rapid Commun. Mass Spectrom.* **18**, 1896–1900 (2004).

23. C. Tokarski, E. Martin, C. Rolando, C. Cren-Olivé, Identification of proteins in renaissance paintings by proteomics. *Anal. Chem.* **78**, 1494–1502 (2006).

24. A. Chambery, A. Di Maro, C. Sanges, V. Severino, M. Tarantino, A. Lamberti, A. Parente, P. Arcari, Improved procedure for protein binder analysis in mural painting by LC-ESI/Q-q-TOF mass spectrometry: Detection of different milk species by casein proteotypic peptides. *Anal. Bioanal. Chem.* **395**, 2281–2291 (2009).

25. D. P. Kirby, M. Buckley, E. Promise, S. A. Trauger, T. R. Holdcraft, Identification of collagen-based materials in cultural heritage. *Analyst* **138**, 4849–4858 (2013).

26. R. Vinciguerra, A. Illiano, A. De Chiaro, A. Carpentieri, A. Lluveras-Tenorio, I. Bonaduce, G. Marino, P. Pucci, A. Amoresano, L. Birolo, Identification of proteinaceous binders in paintings: A targeted proteomic approach for cultural heritage. *Microchem. J.* **144**, 319–328 (2019).

27. M. Mackie, P. Rüther, D. Samodova, F. Di Gianvincenzo, C. Granzotto, D. Lyon, D. A. Peggie, H. Howard, L. Harrison, L. J. Jensen, J. V. Olsen, E. Cappellini, Palaeoproteomic profiling of conservation layers on a 14th century Italian wall painting. *Angew. Chem. Int. Ed.* **57**, 7369–7374 (2018).

28. J. Hendy, F. Welker, B. Demarchi, C. Speller, C. Warinner, M. J. Collins, A guide to ancient protein studies. *Nat. Ecol. Evol.* **2**, 791–799 (2018).

29. J. Hendy, Ancient protein analysis in archaeology. *Sci. Adv.* **7**, eabb9314 (2021).

30. F. Di Gianvincenzo, D. Peggie, M. Mackie, C. Granzotto, C. Higgitt, E. Cappellini, Palaeoproteomics guidelines to identify proteinaceous binders in artworks following the study of a 15th-century painting by Sandro Botticelli's workshop. *Sci. Rep.* **12**, 10638 (2022).

31. C. Warinner, K. Korzow Richter, M. J. Collins, Paleoproteomics. *Chem. Rev.* **122**, 13401–13446 (2022).

32. A. Taurozzi, P. L. Rüther, I. Patramanis, C. Koenig, R. Sinclair Paterson, P. P. Madupe, F. Simon Harking, F. Welker, M. Mackie, J. Ramos-Madrigal, J. V. Olsen, E. Cappellini, Deep-time phylogenetic inference by paleoproteomic analysis of dental enamel. *Nat. Protoc.* **19**, 2085–2116 (2024).

33. J. Mazurek, M. Svoboda, J. Maish, K. Kawahara, S. Fukakusa, T. Nakazawa, Y. Taniguchi, Characterization of binding media in Egyptian Romano portraits using enzyme-linked immunosorbent assay and mass spectrometry. *e-PS* **11**, 76–83 (2014).

34. D. Kirby, M. Svoboda, J. Mazurek, L. R. Spaabæ, J. Southon, Characterization of an unusual coating on funerary portraits from Roman Egypt circa 100-300 AD. *Herit. Sci.* **11**, (2023).

35. C. Brøns, J. Stenger, R. Newman, C. Cartwright, F. Di Gianvincenzo, A. Katerinopoulou, L. Ørsted Brandt, N. Haghipour, L. Hendriks, A lost chapter of ancient art: Archaeometric examinations of panel paintings from Roman Egypt. *Stud. Conserv.* **69**, 557–595 (2024).

36. T. Kasso, J. Stenger, C. Zaggia, G. Pastorelli, M. Ramsøe, T. Ravnsborg, O. N. Jensen, E. Yvanez, C. Spinazzi-Lucchesi, M. J. Collins, C. Brøns, Facing death: A multidisciplinary analysis of a Romano-Egyptian mummy mask at the Ny Carlsberg Glyptotek, Copenhagen. *NPJ Herit. Sci.* **12**, 250 (2024).

37. T. Kasso, C. Zaggia, G. Pastorelli, M. Ramsøe, M. J. Collins, C. Brøns, Behind the mask—Archaeometric analysis of four gilded Romano-Egyptian mummy masks from the Ny Carlsberg Glyptotek, Copenhagen. *J. Archaeol. Sci. Rep.* **64**, 105106 (2025).

38. M. J. Nielsen, M. A. Karsdal, "Chapter 3 - Type III Collagen," in *Biochemistry of Collagens, Laminins and Elastin* (Academic Press, an imprint of Elsevier, 2016).

39. G. Ntasi, S. Sbriglia, R. Pitocchi, R. Vinciguerra, C. Melchiorre, L. Dello Ioio, G. Fatigati, E. Crisci, I. Bonaduce, A. Carpentieri, G. Marino, L. Birolo, Proteomic characterization of collagen-based animal glues for restoration. *J. Proteome Res.* **21**, 2173–2184 (2022).

40. S. Dallongeville, M. Koperska, N. Garnier, G. Reille-Taillefert, C. Rolando, C. Tokarski, Identification of animal glue species in artworks using proteomics: Application to a 18th century gilt sample. *Anal. Chem.* **83**, 9431–9437 (2011).

41. N. E. Robinson, A. Robinson, *Molecular Clocks: Deamidation of Asparaginyl and Glutaminyl Residues in Peptides And Proteins* (Althouse Press, 2004).

42. G. Leo, I. Bonaduce, A. Andreotti, G. Marino, P. Pucci, M. P. Colombini, L. Birolo, Deamidation at asparagine and glutamine as a major modification upon deterioration/aging of proteinaceous binders in mural paintings. *Anal. Chem.* **83**, 2056–2064 (2011).

43. UniProt Consortium, UniProt: A hub for protein information. *Nucleic Acids Res.* **43**, D204–D212 (2015).

44. S. Ikram, *Choice Cuts: Meat Production in Ancient Egypt* (Departement Oosterse Studies, Leuven, Peeters, 1996).

45. H. Epstein, *The Origin of the Domestic Animals of Africa* (Africana Publ. Corp., 1971).

46. A. Janzen, K. Korzow Richter, O. Mwebi, S. Brown, V. Onduso, F. Gatwiri, E. Ndiema, M. Katongo, S. T. Goldstein, K. Douka, N. Boivin, Distinguishing African bovids using zooarchaeology by mass spectrometry (ZooMS): New peptide markers and insights into Iron Age economies in Zambia. *PLOS ONE* **16**, e0251061 (2021).

47. M. Serpico, R. White, "Oil, fat and wax," in *Ancient Egyptian Materials and Technology* (Cambridge Univ. Press, 2000).

48. C. Van Driel-Murray, "Leatherwork and skin products," in *Ancient Egyptian Materials And Technology* (Cambridge Univ. Press, 2000).

49. I. Shaw, P. T. Nicholson, *Dictionary of Ancient Egypt* (British Museum Press, 1995).

50. S. Rossel, F. Marshall, J. Peters, T. Pilgram, M. D. Adams, D. O'Connor, Domestication of the donkey: Timing, processes, and indicators. *Proc. Natl. Acad. Sci. U.S.A.* **105**, 3715–3720 (2008).

51. N. C. Schellmann, Animal glues: A review of their key properties relevant to conservation. *Stud. Conserv.* **52**, 55–66 (2007).

52. D. Bedigian, J. R. Harlan, Evidence for cultivation of sesame in the ancient world. *Econ. Bot.* **40**, 137–154 (1986).

53. D. Q. Fuller, Further evidence on the prehistory of sesame. *Asian Agrihist.* **7**, 127–137 (2003).

54. K. B. Metheny, M. C. Beaudry, *Archaeology of Food: An Encyclopedia*, K. B. Metheny, M. C. Beaudry, Eds. (Rowman & Littlefield, 2015).

55. A. Shevchenko, Y. Yang, A. Knaust, J.-M. Verbavatz, H. Mai, B. Wang, C. Wang, A. Shevchenko, Identification of sesame oil and oil soot ink in organic deposits of Tang Dynasty lamps from Astana necropolis in China. *PLOS ONE* **12**, e0158636 (2017).

56. M. Rageot, R. B. Hussein, S. Beck, V. Altmann-Wendling, M. I. M. Ibrahim, M. M. Bahgat, A. M. Yousef, K. Mittelstaedt, J.-J. Filippi, S. Buckley, C. Spiteri, P. W. Stockhammer, Biomolecular analyses enable new insights into ancient Egyptian embalming. *Nature* **614**, 287–293 (2023).

57. M. S. Copley, H. A. Bland, P. Rose, M. Horton, R. P. Evershed, Gas chromatographic, mass spectrometric and stable carbon isotopic investigations of organic residues of plant oils and animal fats employed as illuminants in archaeological lamps from Egypt. *Analyst* **130**, 860–871 (2005).

58. A. Tchapla, P. Méjanelle, J. Bleton, S. Goursaud, Characterisation of embalming materials of a mummy of the Ptolemaic era. Comparison with balms from mummies of different eras. *J. Sep. Sci.* **27**, 217–234 (2004).

59. J. Łucejko, J. Connan, S. Orsini, E. Ribechini, F. Modugno, Chemical analyses of Egyptian mummification balms and organic residues from storage jars dated from the Old Kingdom to the Copto-Byzantine period. *J. Archaeol. Sci.* **85**, 1–12 (2017).

60. E. Wolff, F. Felber, *Le jardin des pharaons* (Infolio; Musée et jardins botaniques cantonaux, 2016).

61. L. Evans, P. Weinstein, Begone from Me, O Crooked-Lips! Integrated Pest Management in Ancient Egypt. *Am. Entomol.* **67**, 46–53 (2021).

62. L. Manniche, "Perfume," in *UCLA Encyclopedia of Egyptology*, W. Wendrich, Ed. (UCLA Department of Near Eastern Languages and Cultures, 2009).

63. D. B. Redford, *Excavations at Mendes: The Royal Necropolis* (Brill, 2004).

64. J. Ambers, R. Stacey, J. Taylor, Writing that cannot be erased: Investigations of a box of pigmented inlays from the tomb chapel of an Old Kingdom noble. *Br. Museum Technic. Res. Bull.* **1**, 49–54 (2007).

65. M. R. Derrick, D. Stulik, J. M. Landry, *Infrared Spectroscopy in Conservation Science* (Getty Conservation Institute, 1999).

66. M. P. Colombini, F. Modugno, E. Ribechini, "GC/MS in the characterization of lipids," in *Organic Mass Spectrometry in Art and Archaeology* (John Wiley & Sons, Ltd, 2009).

67. S. D. Brent, The production and use of vegetable oils in Ptolemaic Egypt. *Bull. Am. Soc. Papyrol.* **6**, 10.3998/mpub.9749622 (1989).

68. T. Chen, B. Wang, Y. Yang, B. Han, H. Jiang, Sesame use in Turpan during the Tang dynasty: Evidence from the Astana Cemetery. *J. Archaeol. Sci. Rep.* **53**, 104298 (2024).

69. R. H. Wilkinson, *The Complete Gods and Goddesses of Ancient Egypt* (Thames and Hudson, 2003).

70. W. C. Hayes, Inscription from the Palace of Amenhotep III. *J. Near Eastern Stud.* **10**, 156–183 (1951).

71. J. P. Allen, *Genesis in Egypt. The Philosophy of Ancient Egyptian Creation Accounts* (Yale Egyptological Studies 2, 1988).

72. S. Delwen, "Brewing and baking," in *Ancient Egyptian Materials and Technology* (Cambridge Univ. Press, 2000).

73. F. Welc, K. Milecka, L. Marks, Earliest cereal cultivation in Egypt recorded in the Faiyum Oasis lake de posits and its palaeoclimatic context. *Geol. Quart.* **67**, 21 (2023).

74. C. Cennini, C. D. A. Cennini, G. De Beer, *The Craftsman's Handbook*, D. V. Thompson Jr., Ed. (Courier Corporation, 1954), vol. 2.

75. R. Prisby, A. Luchini, L. A. Liotta, C. Solazzo, Wheat-based glues in conservation and cultural heritage: (Dis)solving the proteome of flour and starch pastes and their adhering properties. *J. Proteome Res.* **23**, 1649–1665 (2024).

76. J. Parker, "Analysis of materials used in previous conservation treatments," in *The Nebamun Wall Paintings: Conservation, Scientific Analysis and Display at the British Museum* (Archetype Publications: in association with the British Museum, 2008).

77. O. Duncan, J. Trösch, R. Fenske, N. L. Taylor, A. H. Millar, Resource: Mapping the Triticum aestivum proteome. *Plant J.* **89**, 601–616 (2017).

78. E. Cappellini, L. J. Jensen, D. Szklarczyk, A. Ginolhac, R. A. R. da Fonseca, T. W. Stafford Jr., S. R. Holen, M. J. Collins, L. Orlando, E. Willerslev, M. T. P. Gilbert, J. V. Olsen, Proteomic analysis of a pleistocene mammoth femur reveals more than one hundred ancient bone proteins. *J. Proteome Res.* **11**, 917–926 (2012).

79. B. Demarchi, S. Hall, T. Roncal-Herrero, C. L. Freeman, J. Woolley, M. K. Crisp, J. Wilson, A. Fotakis, R. Fischer, B. M. Kessler, R. R. Jersie-Christensen, J. V. Olsen, J. Haile, J. Thomas, C. W. Marean, J. Parkington, S. Presslee, J. Lee-Thorp, P. Ditchfield, J. F. Hamilton, M. W. Ward, C. M. Wang, M. D. Shaw, T. Harrison, M. Domínguez-Rodrigo, R. D. E.

MacPhee, A. Kwekason, M. Ecker, L. K. Horwitz, M. Chazan, R. Kröger, J. Thomas-Oates, J. H. Harding, E. Cappellini, K. Penkman, M. J. Collins, Protein sequences bound to mineral surfaces persist into deep time. *eLife* **5**, e17092 (2016).

80. J. Cox, M. Mann, MaxQuant enables high peptide identification rates, individualized p.p.b.-range mass accuracies and proteome-wide protein quantification. *Nat. Biotechnol.* **26**, 1367–1372 (2008).

81. S. F. Altschul, W. Gish, W. Miller, E. W. Myers, D. J. Lipman, Basic local alignment search tool. *J. Mol. Biol.* **215**, 403–410 (1990).

82. D. M. Goodstein, S. Shu, R. Howson, R. Neupane, R. D. Hayes, J. Fazo, T. Mitros, W. Dirks, U. Hellsten, N. Putnam, D. S. Rokhsar, Phytozome: A comparative platform for green plant genomics. *Nucleic Acids Res.* **40**, D1178–D1186 (2012).

83. S. Tyanova, T. Temu, J. Cox, The MaxQuant computational platform for mass spectrometry-based shotgun proteomics. *Nat. Protoc.* **11**, 2301–2319 (2016).

84. Y. Perez-Riverol, A. Csordas, J. Bai, M. Bernal-Llinares, S. Hewapathirana, D. J. Kundu, A. Inuganti, J. Griss, G. Mayer, M. Eisenacher, E. Pérez, J. Uszkoreit, J. Pfeuffer, T. Sachsenberg, S. Yilmaz, S. Tiwary, J. Cox, E. Audain, M. Walzer, A. F. Jarnuczak, T. Ternent, A. Brazma, J. A. Vizcaíno, The PRIDE database and related tools and resources in 2019: Improving support for quantification data. *Nucleic Acids Res.* **47**, D442–D450 (2019).

85. A. B. Robinson, J. W. Scotchler, J. H. McKerrow, Rates of nonenzymic deamidation of glutaminyl and asparaginyl residues in pentapeptides. *J. Am. Chem. Soc.* **95**, 8156–8159 (1973).

86. F. Welker, M. A. Soressi, M. Roussel, I. van Riemsdijk, J.-J. Hublin, M. J. Collins, Variations in glutamine deamidation for a Châtelperronian bone assemblage as measured by peptide mass fingerprinting of collagen. *STAR Sci. Technol. Archaeol. Res.* **3**, 15–27 (2017).

87. E. R. Schroeter, T. P. Cleland, Glutamine deamidation: An indicator of antiquity, or preservational quality? *Rapid Commun. Mass Spectrom.* **30**, 251–255 (2016).

88. E. Cappellini, F. Welker, L. Pandolfi, J. Ramos-Madrigal, D. Samodova, P. L. Rüther, A. K. Fotakis, D. Lyon, J. V. Moreno-Mayar, M. Bukhsianidze, R. Rakownikow Jersie-Christensen, M. Mackie, A. Ginolhac, R. Ferring, M. Tappen, E. Palkopoulou, M. R. Dickinson, T. W. Stafford Jr., Y. L. Chan, A. Götherström, S. K. S. S. Nathan, P. D. Heintzman, J. D. Kapp, I. Kirillova, Y. Moodley, J. Agusti, R.-D. Kahlke, G. Kiladze, B. Martínez-Navarro, S. Liu, M. Sandoval Velasco, M.-H. S. Sinding, C. D. Kelstrup, M. E. Allentoft, L. Orlando, K. Penkman, B. Shapiro, L. Rook, L. Dalén, M. T. P. Gilbert, J. V. Olsen, D. Lordkipanidze, E. Willerslev, Early Pleistocene enamel proteome sequences from Dmanisi resolve *Stephanorhinus* phylogeny. *Nature* **574**, 103–107 (2019).

89. D. Han, Z. Yu, J. Lai, C. Yang, Post-translational modification: A strategic response to high temperature in plants. *aBIOTECH* **3**, 49–64 (2022).

90. T. Woodfine, T. Gilbert, "The fall and rise of the scimitar-horned oryx: A case study of ex-situ conservation and reintroduction in practice," in *Antelope Conservation: From Diagnosis to Action*, J. Bro-Jørgensen, D. P. Mallon, Eds. (John Wiley & Sons, 2016), pp. 280–296.

91. V. Linseele, W. Van Neer, S. Thys, R. Phillipps, R. Cappers, W. Wendrich, S. Holdaway, New archaeozoological data from the fayum "Neolithic" with a critical assessment of the evidence for early stock keeping in Egypt. *PLOS ONE* **9**, e108517 (2014).

92. J. Peters, O. Lebrasseur, E. K. Irving-Pease, P. D. Paxinos, J. Best, R. Smallman, C. Callou, A. Gardeisen, S. Trixl, L. Frantz, N. Sykes, D. Q. Fuller, G. Larson, The biocultural origins and dispersal of domestic chickens. *Proc. Natl. Acad. Sci. U.S.A.* **119**, e2121978119 (2022).

93. J. Sigl, Syene II, *Die Tierfunde aus den Grabungen von 2000–2009: Ein Beitrag zur Umwelt—und Kulturgeschichte einer Oberägyptischen Stadt von der Pharaonischen Spät—bis in die Mameluckenzeit* (PeWe-Verlag, 2017).

94. F. N. Hepper, *Pharaoh's Flowers. The Botanical Treasures of Tutankhamun* (KWS Pub., 2009).

95. C. Malleson, R. G. Miracle, "Giza Botanical Database," in *Open Context* (2018); https://doi.org/10.6078/M7JH3J99.

96. M. A. Murray, “Cereal production and processing,” in *Ancient Egyptian Materials and Technology* (Cambridge Univ. Press, 2000).

97. J. Valkoun, J. G. Waines, J. Konopka, “Current geographical distribution and habitat of wild wheats and barley,” in *The Origins of Agriculture and Crop Domestication*, A. B. Damania, J. Valkoun, G. Willcox, C. O. Qualset, Eds. (ICARDA, 1998), pp. 293–299.

98. B. A. Price, B. Pretzel, S. Q. Lomax, Infrared and Raman Users Group Spectral Database, Edition 2007 (Infrared and Raman Users Group; 2014); http://irug.org/search-spectral-database?reset=Reset.
